# Neurokinin-1 receptor drives PKCɑ-AURKA/N-Myc signaling to facilitate the neuroendocrine progression of prostate cancer

**DOI:** 10.1038/s41419-023-05894-x

**Published:** 2023-06-29

**Authors:** Xiao-Wei Zhang, Jing-Yi Li, Lin Li, Wen-Qian Hu, Yan Tao, Wen-Yan Gao, Zi-Nuo Ye, Hao-Yuan Jia, Jia-Nan Wang, Xiao-Kang Miao, Wen-Le Yang, Rui Wang, Ling-Yun Mou

**Affiliations:** 1 School of Life Science Lanzhou University, 222 TianShui South Road, Lanzhou, 730000 P. R. China; 2 Basic Medical Sciences & Research Unit of Peptide Science, Chinese Academy of Medical Sciences, 2019RU066, Lanzhou University, Lanzhou, 730000 China; 3 Key Laboratory of Preclinical Study for New Drugs of Gansu Province, School of Basic Medical Science, Lanzhou University, Lanzhou, 730000 P. R. China; 4 Departemnt of Biochemistry and Molecular Biology, School of basic medical sciences, Fujian Medical University, 1 Xuefu North Road, Fuzhou, 350122 P. R. China; 5 Key Laboratory of Urological Disease of Gansu Province, Lanzhou University Second Hospital, Lanzhou University, Lanzhou, 730000 China

**Keywords:** Tumour biomarkers, Targeted therapies

## Abstract

The widespread application of antiandrogen therapies has aroused a significant increase in the incidence of NEPC, a lethal form of the disease lacking efficient clinical treatments. Here we identified a cell surface receptor neurokinin-1 (NK1R) as a clinically relevant driver of treatment-related NEPC (tNEPC). NK1R expression increased in prostate cancer patients, particularly higher in metastatic prostate cancer and treatment-related NEPC, implying a relation with the progression from primary luminal adenocarcinoma toward NEPC. High NK1R level was clinically correlated with accelerated tumor recurrence and poor survival. Mechanical studies identified a regulatory element in the NK1R gene transcription ending region that was recognized by AR. AR inhibition enhanced the expression of NK1R, which mediated the PKCα-AURKA/N-Myc pathway in prostate cancer cells. Functional assays demonstrated that activation of NK1R promoted the NE transdifferentiation, cell proliferation, invasion, and enzalutamide resistance in prostate cancer cells. Targeting NK1R abrogated the NE transdifferentiation process and tumorigenicity in vitro and in vivo. These findings collectively characterized the role of NK1R in tNEPC progression and suggested NK1R as a potential therapeutic target.

## Introduction

Over 90% of prostate cancer (PCa) are diagnosed as luminal adenocarcinoma featured with abnormal AR expression and activity [1]. Androgen deprivation therapies (ADTs) and AR pathway inhibitors (ARPIs) are the standard care for advanced PCa, however, after temporary regression, tumors relapse is inevitable, and most patients acquire treatment-resistant phenotypes referred to as castration-resistant prostate cancer (CRPC) [2, 3]. The resistance to AR-targeted therapies is associated with alteration of AR status and a considerable subgroup of ARPI-resistant tumors eventually lost their expression of AR and luminal epithelial features, developing into a neuroendocrine phenotype referred to as neuroendocrine prostate cancer (NEPC) [4].

By immunohistochemistry, NEPC is identified with positive expression of neuroendocrine markers like chromogranin A (CgA), enolase 2 (ENO2, neuronal specific enolase), synaptophysin (SYP) and CD56 (NCAM1) as well as negative of AR and other luminal markers [5]. Importantly, compared with epithelial adenocarcinoma, NEPCs exhibit much more malicious characteristics with extensive visceral metastases, poor differentiation, and unfavorable prognosis [6]. Although primary NEPCs are less than 2% of all first-diagnosed PCa, the incidence of treatment-related NEPC (tNEPC) increased up to 17% in castration-resistant patients, who underwent a progression from luminal-type adenocarcinoma to neuroendocrine tumor during antiandrogen treatments [7, 8]. Many studies provided evidence that ARPIs stimulated the evolution of AR-independent mechanisms to fight the survival pressure, despite the initial blocking of luminal adenocarcinoma growth [9]. The molecular mechanisms of tNEPC development driven by ARPIs treatment included oncogenic amplification like aurora A (AURKA) and N-Myc, epigenetic regulation like EZH2 and HP1α as well as activation of transcriptional factors like BRN2 and SOX2 [10–13]. tNEPC tumor cells have also been related to increased cancer cell stemness and epithelial-mesenchymal transition [14, 15]. However, even with these great progresses, NEPC clinical therapy remained unsuccessful and patients have a median overall survival of less than 1 year [16].

Prevalent amplification of neuropeptides and their receptors were reported in about 72% of samples from NEPC patients [17]. Neuropeptides like neurotensin and bombesin were used as NE markers, they exhibited abilities to promote the neuroendocrine transdifferentiation (NEtD) process including acquisition of NE marker expression and acceleration of tumor evolution [18, 19]. Generally, G protein-coupled receptors (GPCR) are the targets recognized by neuropeptides, mediating their intracellular signaling and biological functions [20]. However, the role of neuropeptides and their cognate receptors in NEPCs remains largely unexplored. Substance P (SP) and hemokinin-1 (HK-1) belong to the tachykinin neuropeptide family. In the nervous system, they preferentially bind GPCR neurokinin-1 (NK1R) and regulate physiological functions such as pain transmission, emotion, and vomit reflex [21]. Particularly, NK1R is aberrantly expressed in neural tumors such as glioblastoma and neuroblastoma as well as non-neural tumors like lung cancer and breast cancer. Others and we previously reported NK1R triggered intracellular signaling pathways that contributed to cancer development by facilitating drug resistance, EMT, aberrant cell proliferation, and metastasis [22, 23]. In addition, NK1R was overexpressed in pancreatic intraepithelial neoplasms (PanIN), promoting the oncogenesis of the neoplastic neuroendocrine cells and the PanIN organoid growth [24]. However, few studies have explored the association between NK1R and prostate cancer development.

We analyzed the public sequencing data of prostate cancer patients, finding a significant up-regulation of NK1R expression level in treatment-induced NEPC tumors, which was associated with low AR activity, high NE differentiation, and poor prognosis. These results encouraged us to explore whether NK1R plays a role in tNEPC progression. Our investigation demonstrated that NK1R expression was under the direct regulation of AR. During antiandrogen treatment, the enhanced NK1R expression activates PKC/AURKA/N-Myc pathway in prostate cancer cells, mediating the induction of NE markers and NE-related gene expression and promoting tumor growth. Targeting NK1R by genomic or chemical strategies would inhibit the neuroendocrine transdifferentiation (NEtD) process and tumorigenicity in vitro and in vivo.

## Results

### NK1R expression is correlated with Gleason grade and unfavorable prognosis

**Fig. 1:**
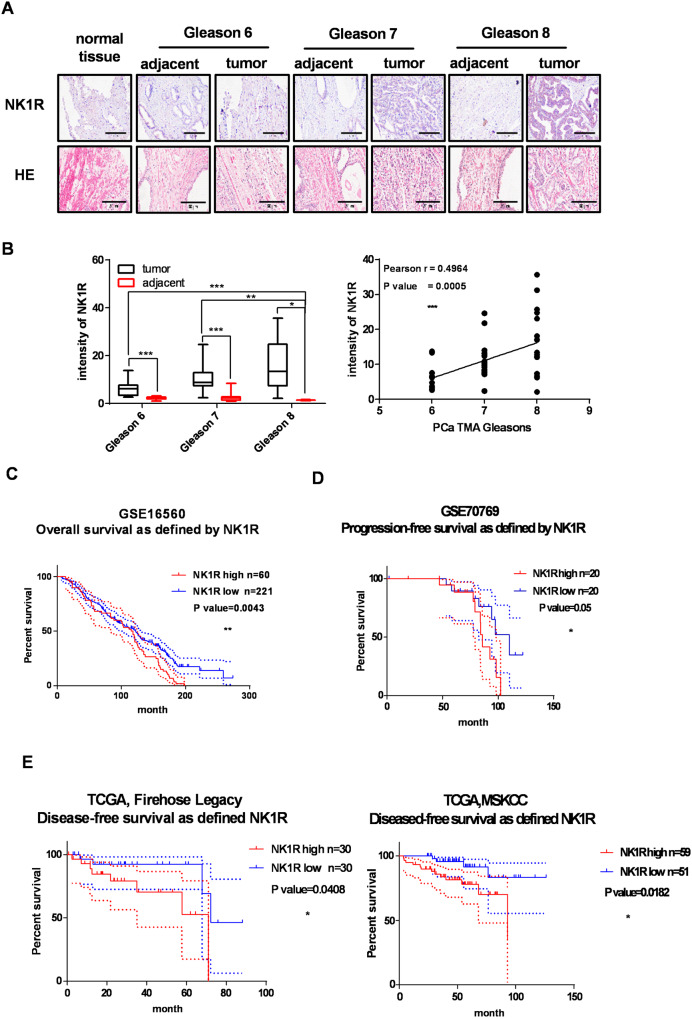
NK1R expression is clinically correlated with Gleason grade and unfavorable prognosis. **A** IHC analysis of NK1R expression in human prostate tumor TMA compared with matched adjacent tissues. Gleason 6: *n* = 20; Gleason 7: *n* = 60; Gleason 8: *n* = 10; Normal tissue: *n* = 3; Scar bar, 50 μm. **B** IHC staining intensity of NK1R in human prostate tumor TMA. Left: human prostate tumor tissues compared with matched adjacent tissues; right: the correlation between NK1R expression and Gleason grade. The Kaplan–Meier analysis of disease progression in prostate cancer patients is defined by NK1R expression level (low/high), **C** Overall survival; **D** progression-free survival; **E** disease-free survival. *P < 0.05, **P < 0.01, ***P < 0.001.

To determine whether NK1R is clinically relevant to human PCas, we performed IHC on tissue microarrays (TMA) containing cancer and adjacent normal tissues from 90 cases of PCa patients. As shown in Fig. 1A, B, NK1R protein level was elevated in tumor tissues compared with the adjacent tissues; the intensity of NK1R staining was highest in PCas with a Gleason score 8. We further analyzed the association of NK1R with PCa patients’ prognosis. A cohort of 281 PCa cases from GSE16560 [25] were divided into two groups according to their NK1R expression level (NK1R^low^ and NK1R^high^). NK1R^low^ group showed a better prognosis with overall survival of 122 months lifetime than the NK1R^high^ group (116 months, *p* = 0.0043) (Fig. 1C). Progression-free or disease-free survival was assessed in three independent published studies. In PCa patients who accepted robotic radical prostatectomy surgery (GSE70769) [26], biochemical relapse occurred in the NK1R^high^ group in 85.7 months after the first surgery, while the NK1R^low^ group occurred in 110.2 months (*p* = 0.05) (Fig. 1D). Disease-free survival was assessed in patients of Firehose Legacy and MSKCC from TCGA [27]. High NK1R expression was associated with accelerated tumor relapse in the single-variation model (*p* = 0.0182, Fig. 1E). These results demonstrated NK1R expression level was associated with high Gleason score and unwelcome tumor recurrence in PCa patients.

### NK1R is co-upregulated with NE markers and NE-related genes in tNEPC

**Fig. 2:**
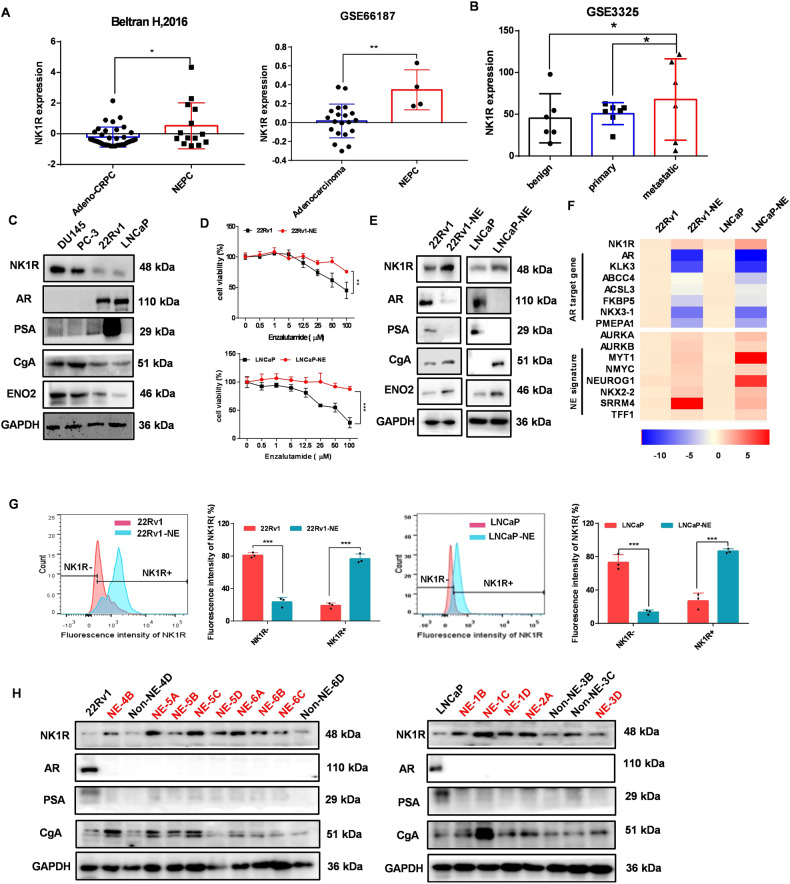
NK1R is a cell surface receptor co-upregulated with NE markers and NE-related genes in tNEPC. **A** Comparison of NK1R expression in human CRPCs (*n* = 34) and NEPCs (*n* = 14) from the dataset of Beltran H et al. 2016 or in human prostate adenocarcinomas (*n* = 20) and NEPCs (*n* = 4) (GSE66187). **B** NK1R expression in benign prostate tissues (*n* = 6), localized primary prostate cancer tissues (*n* = 7), and metastatic prostate cancer tissues (*n* = 6) (GSE3325). **C** Protein level of CgA, ENO2, AR, PSA, and NK1R in a panel of prostate cancer cell lines. **D** Cell survival of 22Rv1-NE, LNCaP-NE, and their parental cells treated with enzalutamide for 72 h. **E** Protein level of NK1R is upregulated in enzalutamide-resistant 22Rv1 and LNCaP cells compared to parental 22Rv1 and LNCaP cells. **F** Relative mRNA level of NE-related genes and AR target genes in 22Rv1-NE, LNCaP-NE, and their parental cells. **G** FACS analysis and quantification of NK1R expression on the cell surface of 22Rv1, 22Rv1-NE, LNCaP, and LNCaP-NE cells. **H** Protein level analysis of NK1R, AR, PSA, and CgA in sub-colonies derived from 22Rv1-NE and LNCaP-NE cells. Data was presented as mean ± SD, *P < 0.05, **P < 0.01, ***P < 0.001.

We evaluated the expression of NK1R and its ligands in RNA-Seq data of Beltran et al. cohort including CRPC adenocarcinoma and CRPC-derived tNEPC patient samples [28]. Compared with CRPC adenocarcinoma patients, NK1R expression was significantly upregulated along with the expression of NE marker CgA, SYP, and the genes related to neuroendocrine and nervous system development including MYT1, SRRM4, etc. (referred to as NE-related genes) in CRPC-derived tNEPC patients, while the adenocarcinoma marker AR and AR target genes like KLK3 and ABCC4 were low (Fig. 2A and Fig. S1A). The elevation of NK1R expression was also demonstrated in LuCaP patient-derived xenograft (PDX) models [29]. In the RNA-Seq data of GSE66187, NK1R level was significantly higher in the neuroendocrine PDXs than in the adenocarcinoma LuCaP PDX models (Fig. 2A and Fig. S1B). In addition, in comparison with benign or primary PCas, NK1R was highest in metastatic PCas, accompanied with increased expression of NE markers (CgA/B, ENO2, and SYP) and NE-related genes such as ADAM22, MYT1, and SRRM4 (GSE3325) [30] (Fig. 2B and Fig. S1C).

We further examined the expression of NK1R and NE markers in a panel of human PCa cell lines. NK1R was highly expressed in NE-like cell lines PC-3 and DU145, both positive of NE markers (CgA and ENO2) and negative of AR/PSA; in contrast, in AR-positive prostate adenocarcinoma cell line LNCaP and 22Rv1, NK1R expression is much lower and NE markers are marginal (Fig. 2C). We established tNEPC cell models by recapitulating ARPI treatment in LNCaP and 22Rv1 cells. After 6 months of treatment with enzalutamide, both cell lines exhibited complete ENZ-resistance (Fig. 2D), the expression of NE markers and NE-related genes significantly increased, and the expression of AR and KLK3 (encoding PSA) were strongly inhibited, suggesting the occurrence of typical NEtD process. We named the derived cell lines as LNCaP-NE and 22Rv1-NE respectively. A remarkable increase of NK1R expression was detected in both tNEPC cell models (Fig. 2E, F). FACS analysis confirmed that the percentage of cells with positive NK1R expression on the cell surface was significantly augmented up to 80% in LNCaP-NE and 22Rv1-NE cells (Fig. 2G). Single-cell clone analysis demonstrated that all clones showed an absence of AR and KLK3 expression, while about 75% of them were NK1R positive. Notably, all NK1R-positive clones demonstrated high expression of NE marker CgA but not the NK1R-negative clones (Fig. 2H). These data suggested a positive association of NK1R expression with tNEPC patients and that NK1R expression increased in the NEtD process related to AR inhibitor administration.

### AR directly blocks the expression of NK1R in AdPC cells

**Fig. 3:**
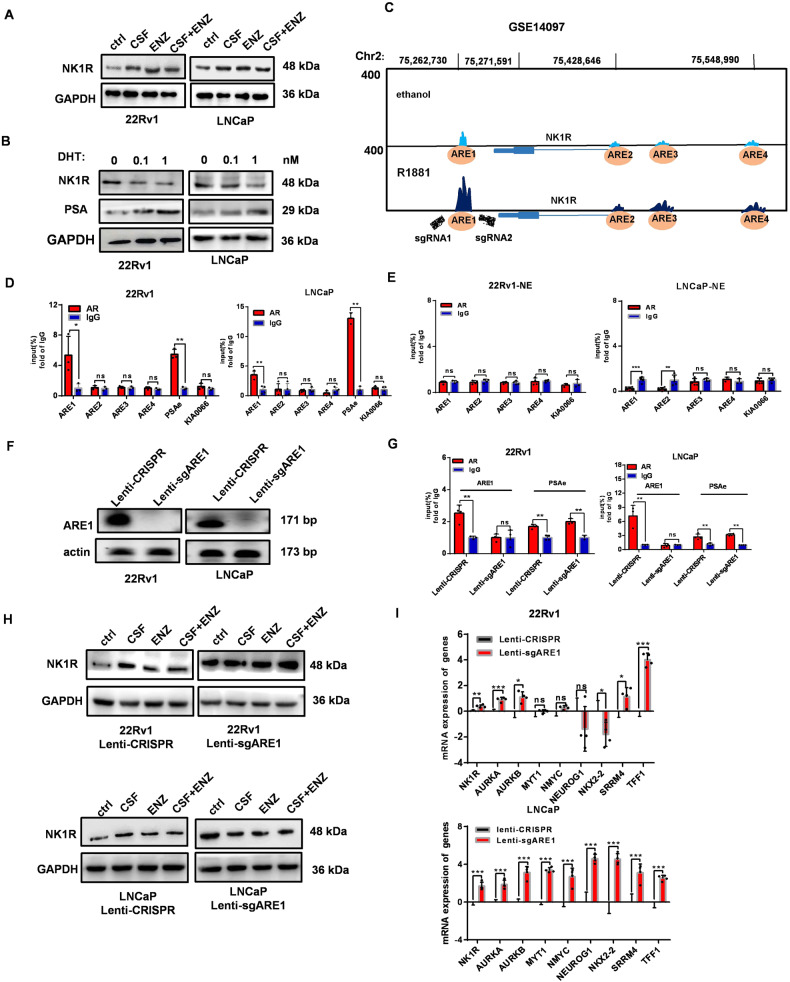
AR directly blocks the expression of NK1R in AdPC cells. **A** Protein level of NK1R in 22Rv1 and LNCaP cells treated with ENZ and/or CSF for 1 week. **B** CHIP-Seq data (GSE14097) in hormone-starved LNCaP cells treated with ethanol or R1881 is re-analyzed and ChIP-SEQ signal around NK1R gene is indicated (ARE1–4). **C** Protein level of NK1R in 22Rv1 and LNCaP cells treated with DHT (0–1 nM) for 36 h. **D** ChIP of AR antibody was performed in 22Rv1 and LNCaP cells which are pre-incubated in a medium with 10% CSF for 1 week and then treated with 1 nM DHT for 36 h; QPCR was performed with primers flanking NK1R gene promoter or enhancer regions (ARE1–4). IgG is used as a negative control antibody. **E** ChIP-QPCR of AR antibody was performed in 22Rv1-NE and LNCaP-NE cells with primers flanking the potential ARE sites of NK1R gene expression regulatory regions (ARE1–4) as described. **F** Genomic DNA PCR for CRISPR-Cas9 mediated ARE1 deletion in 22Rv1 and LNCaP. Cells were transfected with plasmids of Lenti-sgARE1 or control Lenti-CRISPR for 48 h and harvested for genomic DNA PCR. ACTIN was used as a loading control. **G** ChIP-QPCR of AR antibody was performed in 22Rv1 and LNCaP cells transfected with plasmids of Lenti-sgARE1 or control Lenti-CRISPR with primers flanking the ARE1 region as described. **H** Protein level of NK1R in 22Rv1 and LNCaP cells transfected with plasmids of Lenti-sgARE1 or control Lenti-CRISPR. Cells were treated with or without ENZ and/or CSF for 1 week and the whole cell lysates were analyzed by Western blot. **I** Expression of NK1R and NE signature genes in 22Rv1 and LNCaP cells transfected with plasmids of Lenti-sgARE1 or control Lenti-CRISPR. Data were shown as mean ± SD, *P < 0.05, **P < 0.01, ***P < 0.001.

AR usually exerted its functions as a transcriptional regulator of specific target genes [10, 31, 32]. As shown in Fig. 3A, B, in 22Rv1 and LNCaP cells, NK1R expression was induced under AR antagonism condition for 7 days, while reactivating AR with DHT significantly reduced NK1R level. To investigate the underlying mechanisms, we analyzed published AR CHIP-Seq data (GSE14097) [33]. There is a potential AR response element binding site at 9 kb downstream of NK1R gene transcription ending site (ARE1) as well as three others (ARE2-4) in promoter region respectively (Fig. 3C). CHIP-QPCR assay was performed in LNCaP and 22Rv1 cells with AR antibody, showing that with DHT treatment, AR was remarkably recruited at ARE1 site in both cell lines but much less at ARE2-4 sties (Fig. 3D). In contrast, the recruitment of AR at ARE1 was not detected in LNCaP-NE and 22Rv1-NE cells, both negative of AR (Fig. 3E). To confirm the significance of ARE1 binding site in regulating NK1R expression, we transfected cells with sgRNA flanking the ARE1 binding site in CRISPR-Cas9 lentivirus (Fig. S2). PCR analysis validated a CRISPR-Cas9 mediated deletion of genomic DNA (Fig. 3F). CHIP-QPCR result showed that AR recruitment to the ARE1 site was significantly reduced after ARE1 deletion while the canonical AR target gene PSA was not affected (Fig. 3G). Importantly, ARE1-deletion reinstated NK1R expression in LNCaP and 22Rv1 cells and AR inhibition could not additionally stimulate the increase of NK1R expression (Fig. 3H); moreover, ARE1-deletion-induced NK1R expression was accompanied by enhanced level of NE-related genes such as *AURKA* and *SRRM4* (Fig. 3I). These data suggested that AR negatively regulated NK1R expression by directly binding the ARE1 site and the loss of AR expression/activity would release the blockade and restore NK1R expression.

### NK1R drives the development of NE phenotype and therapeutic resistance

**Fig. 4:**
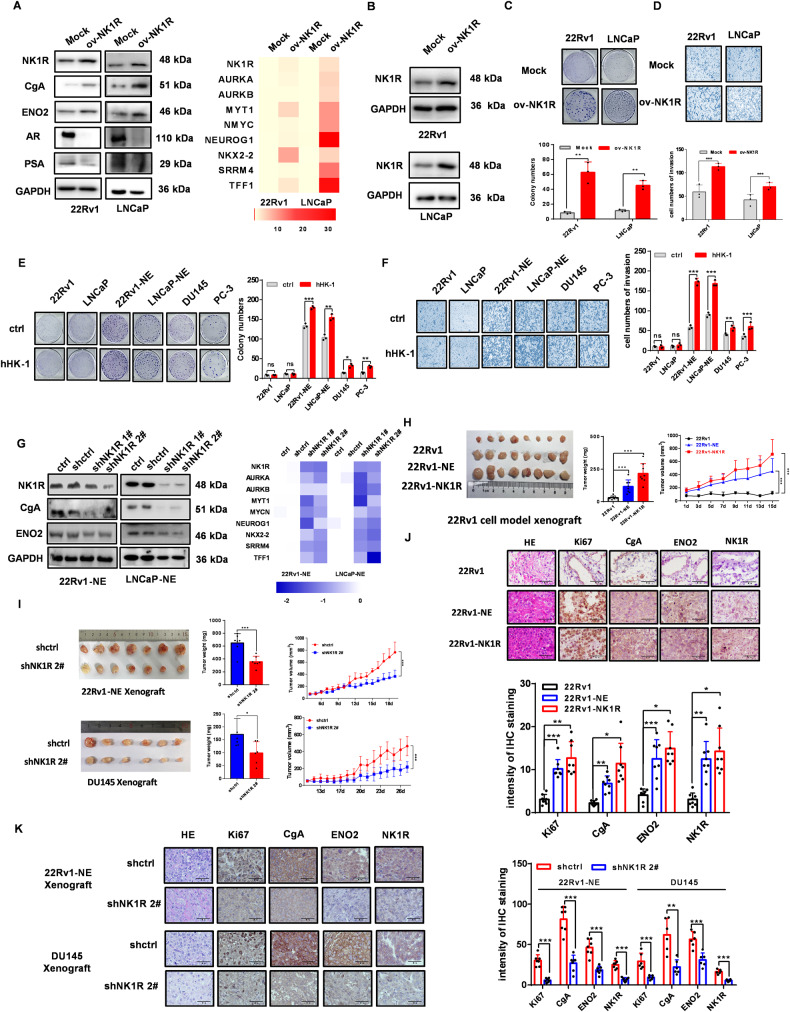
NK1R drives the development of NE phenotype and therapeutic resistance. **A** The effect of NK1R overexpression on CgA, ENO2, AR, and PSA (left) and NE signature genes expression (right) in 22Rv1 and LNCaP. **B** Western blot analysis of NK1R expression in cells transfected by plenti-EGFP-Puro-CMV-NK1R (ov-NK1R) or control plasmid (Mock). Up: 22Rv1; down: LNCaP. GAPDH was used as control. **C** The effect of NK1R overexpression on colony formation, colonies were calculated with Image J (down). **D** The effect of NK1R overexpression on transwell cell invasion in 22Rv1 and LNCaP cells, the infiltrated cells were calculated with Image J (down). **E** The effect of NK1R activation by 1 μM hHK-1 on colony formation, colonies were calculated with Image J (right). **F** The effect of NK1R activation by 1 μM hHK-1 on transwell cell invasion in DU145/PC-3, 22Rv1/LNCaP, and 22Rv1-NE and LNCaP-NE cells, the infiltrated cells were calculated with Image J (right). **G** The effect of shRNA-mediated NK1R knockdown on CgA, ENO2 (left), and NE signature genes expression (right) in 22Rv1-NE and LNCaP-NE. **H** Tumor volume and weight of 22Rv1, 22Rv1-NE, and 22Rv1-NK1R xenografts grown in nude mice (*n* = 8). **I** Tumor volume and weight of 22Rv1-NE, 22Rv1-NE-shNK1R2#, DU145, and DU145-shNK1R2# xenografts grown in nude mice (*n* = 7). **J** IHC staining of NK1R, Ki67, CgA, ENO2 expression in 22Rv1/22Rv1-NK1R/22Rv1-NE xenografts. Scar bar, 50 μm. The intensity of relative protein was calculated with Image Pro Plus (down). **K** IHC staining of NK1R, Ki67, CgA, ENO2 expression in 22Rv1-NE/22Rv1-NE-shNK1R2# (up) and DU145/DU145-shNK1R2# xenografts (down). Scar bar, 50 μm. The intensity of relative protein was calculated with Image Pro Plus (right). Data were shown as mean ± SD, *P < 0.05, **P < 0.01, ***P < 0.001.

To address whether NK1R is a driver of the NE molecular features and phenotypes development, we overexpressed NK1R in LNCaP and 22Rv1 cells. LNCaP-NK1R and 22Rv1-NK1R demonstrated significant expression of NE marker CgA and ENO2 as well as the NE-related gene while the level of adenocarcinoma marker AR and PSA reduced (Fig. 4A and Fig. S3A). Besides the alteration of lineage markers, LNCaP-NK1R and 22Rv1-NK1R cells exhibited aggressive phenotypes, with accelerated cell proliferation, colony formation, and invasion (Fig. 4B–F and Fig. S3B, C). They became resistant to enzalutamide treatment even these cells were treatment-naive (Fig. S3D). In contrast, after transfecting NK1R-specific shRNA in LNCaP-NE and 22Rv1-NE cells, the loss of NK1R significantly reduced the level of NE markers as well as NE-related genes (Fig. 4G). Similar results were observed in NE-like DU145 and PC-3 cells (Fig. S3E).

We then evaluated the role of NK1R in tumorigenesis in a xenograft model. In nude Bal/BC mice, tumors initiated by 22Rv1-NE or 22Rv1-NK1R grew notably faster and bigger than by 22Rv1 (Fig. 4H). In contrast, NK1R knockdown significantly delayed the growth of 22Rv1-NE and DU145 xenografts with reduced tumor weight and volume (Fig. 4I). IHC staining showed that 22Rv1-NE and 22Rv1-NK1R grafts were with much more significant expression of NE markers CgA and ENO2 along with a higher level of proliferative marker Ki67 (Fig. 4J). On the contrary, NK1R knockdown reduced the expression of CgA and ENO2 as well as Ki67 in the xenografts of 22Rv1-NE and DU145 (Fig. 4K and Fig. S3F). These results, taken together, suggest that elevated NK1R expression can turn on the NEtD process in adenocarcinoma prostate cancer cells, promoting their transition into the neuroendocrine lineage and accelerating their cell survival, growth, and therapy resistance.

### NK1R regulates the AURKA-N-Myc pathway in tNEPC cells

**Fig. 5:**
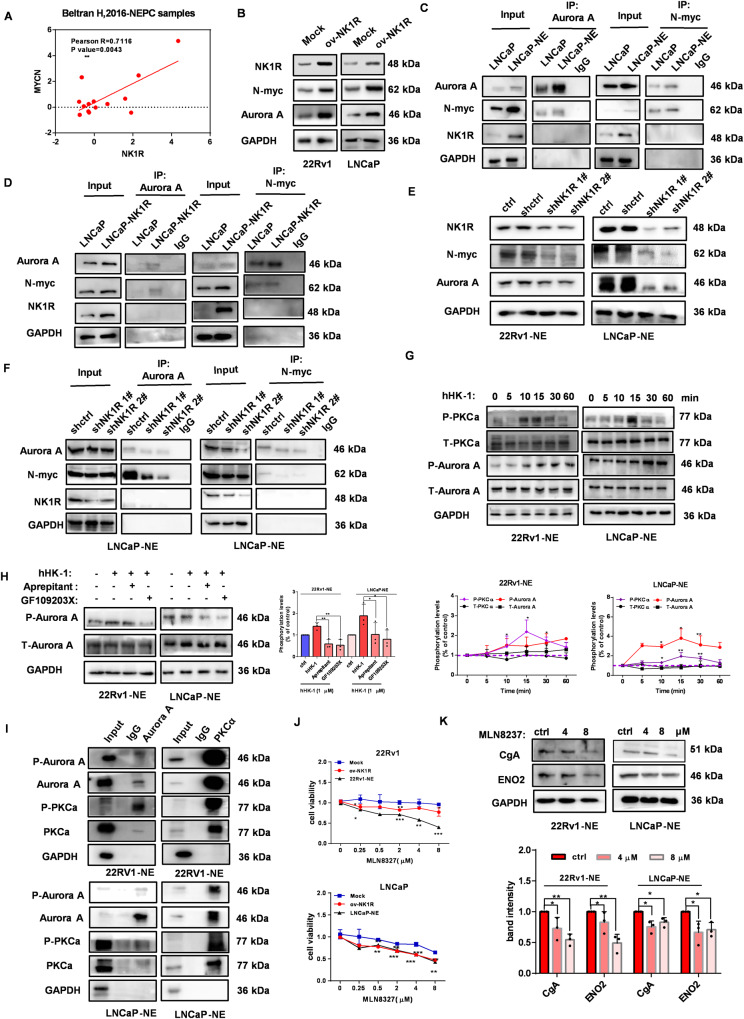
NK1R regulates the AURKA-N-Myc pathway in tNEPC cells. **A** Correlation analysis of NK1R expression and N-Myc expression in tNEPC patients. **B** The effect of NK1R overexpression on N-Myc and AURKA expression in 22Rv1 and LNCaP. **C**, **D** Co-IP assay by the antibodies of N-Myc or AURKA to detect AURKA/N-Myc interaction in LNCaP-NE (**C**) /LNCaP-NK1R (**D**) cells. **E** The effect of shRNA-mediated NK1R knockdown on N-Myc and AURKA expression in 22Rv1-NE and LNCaP-NE. **F** The effect of NK1R knockdown on AURKA/N-Myc interaction detected by Co-IP assay. IgG was used as a negative control antibody. **G** hHK-1 induced PKCα and AURKA phosphorylation in 22Rv1-NE and LNCaP-NE cells in a time-dependent way by western blot (left). Band intensity was calculated with Image J and normalized by control (down). **H** AURKA phosphorylation level was reduced by Aprepitant and GF109203X treatment in 22Rv1-NE and LNCaP-NE cells. The cells were pre-treated with aprepitant (2 μM) or GF109203X (1 μM) for 30 min and stimulated by 1 μM hHK-1 for 30 min before subjecting to western blot analysis. The band intensity was shown by Image analysis and normalized by control (right). **I** Co-IP assay by the antibodies of PKCα or AURKA to detect PKCα-AURKA interaction in 22Rv1-NE/LNCaP-NE cells. **J** Cell survival assay in 22Rv1, 22Rv1-NE/NK1R and LNCaP, LNCaP-NE/NK1R cells. Cells were treated with MLN8237 at indicated concentrations for 72 h. **K** MLN8237 reduced the protein level of CgA and ENO2 in 22Rv1-NE and LNCaP-NE cells. Cells were treated with MLN8237 for 24 h and the whole cell lysates were analyzed by western blot. Band intensity was calculated by Image J and normalized by GAPDH. Data were shown as mean ± SD, *P < 0.05, **P < 0.01, ***P < 0.001.

N-Myc overexpression is common in NEPC and destabilization of N-Myc by AURKA inhibition reduces tumor burden [34, 35]. It is noteworthy that N-Myc expression was strongly positively related to NK1R in CRPC-derived NEPC patients of the Beltran et al. cohort (Fig. 5A). Compared with adenocarcinoma cell LNCaP and 22Rv1, both AURKA and N-Myc protein level were much higher in LNCaP-NE and 22Rv1-NE as well as in PC-3/DU145 (Fig. S4A, B). Notably, NK1R overexpression significantly enhanced the level of AURKA and N-Myc in LNCaP and 22Rv1 (Fig. 5B). We furthermore performed a Co-IP assay and found a substantial protein-protein interaction between AURKA and N-MYC in LNCaP-NE and 22Rv1-NE cells as well as in 22Rv1-NK1R and LNCaP-NK1R (Fig. 5C, D and Fig. S4C, D). Although NK1R was not involved in the protein complex, NK1R knockdown reduced AURKA and N-Myc expression levels and thus alleviated their interaction (Fig. 5E, F and Fig. S4E).

Except for stabilizing the N-Myc protein, AURKA is also an important regulator of mitosis and cell cycle progression [36]. Interestingly, we found that hHK-1 triggered significant phosphorylation of AURKA in LNCaP-NE, 22Rv1-NE, DU145, and PC-3 (Fig. 5G and Fig. S4F), which was inhibited by NK1R antagonist aprepitant (Fig. 5H and Fig. S4G). PKC is a key component in the NK1R-mediated signaling pathway, which has been reported to be responsible for AURKA phosphorylation in tumor and neural cells [37, 38]. Western blot showed that activation of NK1R induced a time-dependent phosphorylation of PKCα (Fig. 5G and Fig. S4F). PKC inhibitor GF109203X was able to reduce NK1R-mediated AURKA phosphorylation and induce cell cycle G2/M arrest (Fig. 5H and Fig. S4G, H). Western blot demonstrated that AURKA could be co-precipitated with PKCα and vice versus in LNCaP-NE and 22Rv1-NE cells (Fig. 5I), similar results were detected in DU145, PC-3, 22Rv1-NK1R and LNCaP-NK1R (Fig. S4I). These results suggested NK1R-mediated PKC signaling was involved with the regulation of AURKA phosphorylation in tNEPC cells.

AURKA inhibitor has been applied as a therapeutic strategy of NEPC in clinical trials [39]. We used a selective AURKA inhibitor MLN8237 to treat the tNEPC cells in vitro. In comparison with 22Rv1 and LNCaP cells, 22Rv1-NE and LNCaP-NE were much more sensitive to MLN8237; meanwhile, overexpression of NK1R in 22Rv1 and LNCaP significantly increased their sensitivity to MLN8237 treatment (Fig. 5J). In accordance with the previous reports, MLN8237 reduced the interaction of N-Myc and AURKA, decreasing the level of CgA and ENO2 in 22Rv1-NE/LNCaP-NE cells (Fig. 5K and Fig. S4J, K). Similar effects were in LNCaP-NK1R/22Rv1-NK1R and DU145/PC-3 (Fig. S4J, K). Taking these data together, we concluded that NK1R enhanced the signaling of the AURKA-N-Myc pathway in tNEPC cells, promoting the development of NE features and tumor growth in vitro.

### Inhibition of NK1R blocks the growth of ENZ-resistant and NE-like CRPC in vitro and vivo

**Fig. 6:**
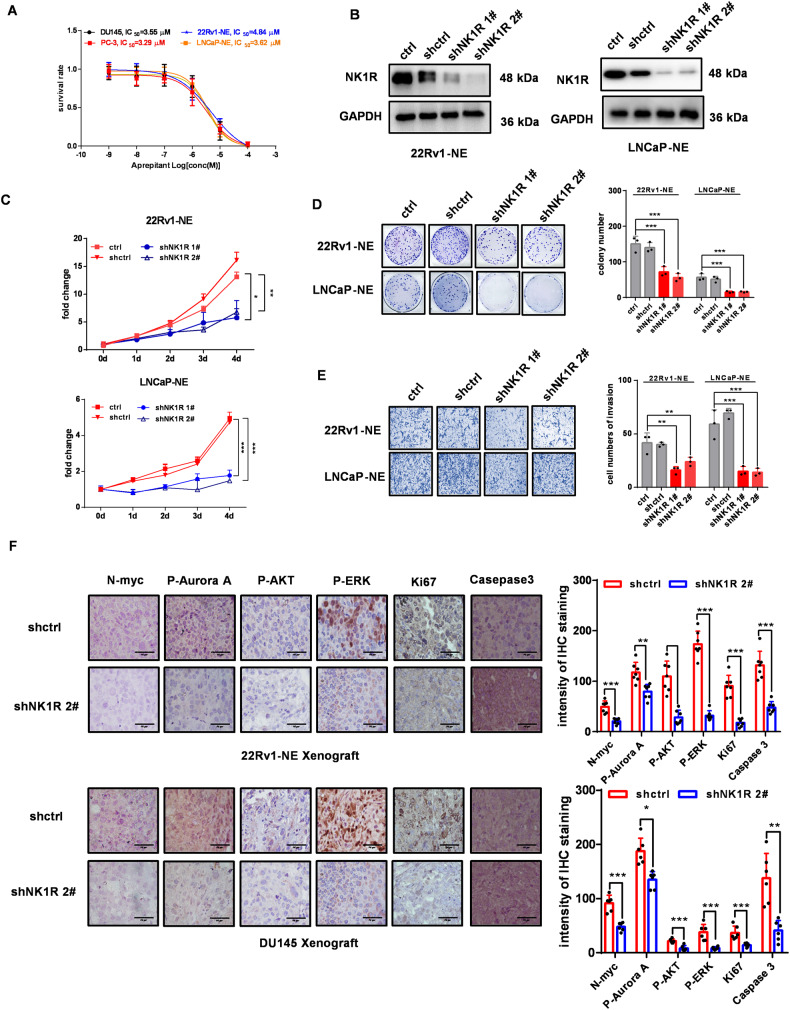
Inhibition of NK1R blocks the growth of ENZ-resistant and NE-like CRPC in vitro and vivo. **A** IC50 values of aprepitant and enzalutamide in various prostate cancer cell lines. **B** Western blot assay of shRNA-mediated NK1R knockdown in 22Rv1-NE and LNCaP-NE cells. The effect of NK1R knockdown on **C** cell growth curve, **D** colony formation, and **E** transwell cell migration. Colonies and the infiltrated cells were calculated by Image J. **F** IHC analysis of N-myc, p-AURKA, p-ERK, p-AKT, Ki67, and Caspase3 in tumor xenografts. Scar bar, 50 μm. Data were shown by mean ± SD, *P < 0.05, **P < 0.01, ***P < 0.001.

We further investigated whether NK1R would be an effective target for tNEPC therapy. The selective antagonist of NK1R aprepitant is used in clinics to prevent chemo-induced nausea and vomiting [40]. LNCaP-NE and 22Rv1-NE were resistant to enzalutamide treatment (Fig. 2D), but the aprepitant showed high efficiencies to inhibit their cell proliferation. Aprepitant also significantly reduced the cell survival ability of NE-like cell lines PC-3 and DU145, which were insensitive to enzalutamide (Fig. 6A). Consistently, NK1R knockdown markedly reduced the proliferation and invasion of LNCaP-NE and 22Rv1-NE cells (Fig. 6B–E) as well as in DU145 and PC-3 (Fig. S5A–C). In addition, the antitumor effect of docetaxel and paclitaxel was significantly enhanced in cells when NK1R was knocked down (Fig. S5D). In xenograft tumors of 22Rv1-NE and DU145, IHC staining showed that NK1R knockdown was disadvantageous for cell survival and proliferation, indicated by remarkable attenuation of Ki67, p-AURKA, N-Myc, p-ERK, and p-AKT as well as the increase of Caspase3 level (Fig. 6F). These data showed that targeting NK1R, alone or combined with chemotherapy, would be a potential strategy to treat tNEPC which is resistant to current antiandrogen therapeutics.

## Discussion

**Fig. 7:**
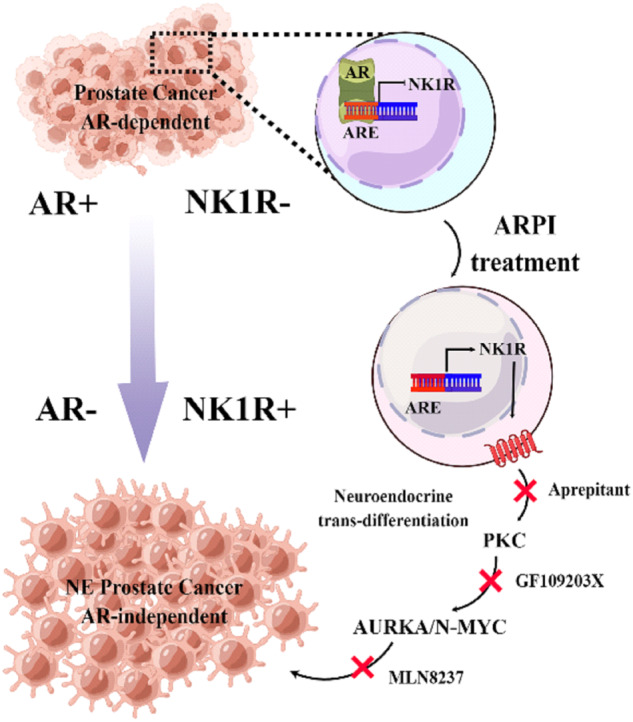
Work model of NK1R regulated NE progression by PKC-AURKA/N-Myc pathway.

Most current clinical therapies target AR-positive luminal-type tumor cells in prostate cancer; however, these treatments, either ADT or ARPIs, eventually failed because they induced lineage plasticity progression in prostate adenocarcinoma and greatly increased the number of non-luminal cells like NE-type tumor cells [41]. Compared with luminal tumor cells, NE tumor cells demonstrated extraordinary aggressiveness and little response to chemotherapies and ARPIs [42]. In the current study, we identified a cell surface receptor NK1R as a novel clinically relevant driver of tNEPC. Antiandrogen therapies significantly induce the expression of NK1R, which further contribute to NEtD, aggressive tumor growth, and drug-resistance in prostate cancer cells. Notably, inhibition of NK1R would show abilities to block prostate cancer progression by impeding the process of NEtD and tumor growth in vitro and in vivo (Fig. 7).

Besides the expression of NE markers like CgA and ENO2, the NE cell population are also featured with the expression of neuropeptide receptors such as bombesin, neurotensin, and calcitonin receptor [18, 19, 43, 44]. NK1R is predominantly expressed in the central and peripheral nervous system, where it plays physiological roles like pain transmission, emotion, and vomit reflex [45, 46]. In addition, abnormal NK1R level is identified in neural tumor such as glioblastoma, neuroblastoma [47], and melanoma [48] in which it mediates the intracellular signaling pathways of cell proliferation, invasion, and survival. NK1R is expressed in various stages of prostate cancer [49]. NK1R was also confirmed to express in prostate cancer cells compared to normal cells in human fetal foreskin fibroblast cells (HFF-1) [50]. RNA-sequencing data from patient cohorts showed that NK1R expression was identified in both CRPC adenocarcinoma and CRPC-derived NEPC; however, compared with CRPC adenocarcinoma, NK1R was significantly higher in CRPC-derived NEPC (Fig. 2A). Additionally, in comparison with primary prostate cancer, NK1R was highest in metastatic prostate cancer patients, accompanied with increasing expression of NE markers and NE-related genes (GSE3325) [30] (Fig. 2B). It is worthy of note that NK1R expression was inversely correlated with the expression of AR or AR-targeted genes in these patient-derived samples (Fig. S1B–D). We also found that in both adenocarcinoma tumor cells and PDX models, NK1R expression was inducible with AR inhibitor or castration treatment (Fig. 2C–F). These results suggested that NK1R was not a specific marker of NEPC, but it may serve as a potential marker indicating the progression from luminal-type adenocarcinoma toward NEPC, especially under the circumstance of AR-targeted therapies. Mechanical studies in cell models demonstrated that NK1R expression was inhibited by AR in a ligand-dependent way. An ARE site was identified at the downstream transcription ending site of the NK1R gene; when activated by DHT, AR occupied the site and inhibited NK1R expression. Either loss of AR expression/activity or deletion of the ARE1 sequence can restore NK1R expression in prostate cancer cells (Fig. 3). These results suggested that the elevation of NK1R was an adaptive response of prostate adenocarcinoma to antiandrogen therapies: during AR-targeted therapies, the loss of AR activity released the impediment and restored the expression of NK1R, then enhancing NK1R expression level in prostate cancer cells.

We demonstrated that elevated NK1R played important roles in driving the NE lineage plasticity in prostate adenocarcinoma and facilitating tumor cell proliferation, invasion, and resistance to ARPI treatment. NK1R-positive cells are highly populated in tNEPC models with significant expression of classical NE markers and NE-development-related genes (Fig. 2C–H). These cells were much more aggressive in comparison with their parental adenocarcinoma cells, showing ligand-dependent proliferation, colony formation, and invasion via NK1R activation (Fig. 4E, F and Fig. S2C). Interestingly, when examining the sub-clones from ENZ-treatment-induced NEPC cells, we found that although all detected clones were absent of AR expression, only those with positive expression of NK1R displayed acquisition of terminal NE markers (Fig. 2G). This result was in accordance with the idea that not all prostate cancer lacking AR function would evolve into an NEPC [51] and shed light on the importance of NK1R in driving the NEtD of prostate cancer cells. Overexpression of NK1R in epithelial prostate cancer cells was able to induce a shift of lineage plasticity, leading to the reduction of AR and PSA levels, the gain of the expression of CgA, ENO2, AURKA, N-Myc, MYT1, and SRRM4 (Fig. 4A and Fig. S3A). Even though these cells were treatment-naïve, they were resistant to enzalutamide treatment, becoming highly proliferative and invasive (Fig. 4B–D and Fig. S3B, D).

The amplification of AURKA and N-Myc is frequent in NEPCs [34]. The deregulation of N-Myc shows a causal relationship with tNEPC development by directly promoting the transcription of NE markers and DNA damage-response pathway genes, while AURKA can stabilize N-Myc by inhibiting the interaction of N-Myc with the E3 ubiquitin ligase [35]. We found that overexpression of NK1R enhanced both AURKA and N-Myc level as well as their protein-protein interaction (Fig. 5B–D and Fig. S4C, D). Besides stabilization of the N-Myc protein, AURKA also promotes tumorigenesis by phosphorylating the protein substrates, some of which are mediators of uncontrolled mitosis and cell cycle progression mitosis [52]. When examining NK1R-mediated signaling pathways, we showed for the first time that NK1R could trigger AURKA phosphorylation at Thr288 in tNEPC cells (Fig. 5G and Fig. S4F). NK1R-mediated PKCα activation was involved with the phosphorylation of AURKA because AURKA was co-immunoprecipitated with PKCα and the inhibition of PKC with GF109203X reduced the AURKA phosphorylation mediated by NK1R in tNEPC cells (Fig. 5H, I and Fig. S4G, I). Notably, AURKA inhibitor MLN8237 showed a significant anti-proliferation effect in 22Rv1-NE and LNCaP-NE cells while reducing the NE marker CgA and ENO2 expression (Fig. 5J, K). Interestingly, overexpressing NK1R in 22Rv1 and LNCaP cells increased their sensitivity to MLN8237 (Fig. 5J). Although further investigation would be necessary to decode the precise mechanisms, these data implied a mechanism of NK1R-PKC-AURKA-N-Myc signaling axis, which regulated the NEtD process and uncontrollable tumor growth.

Since NK1R expression was associated with the NEtD process and accelerated tumor relapse and the NEtD process, we did a preclinical evaluation of whether NK1R inhibition could be an optional strategy for tNEPC treatment. Aprepitant is the selective antagonist of NK1R and is approved by FDA for the treatment of chemotherapy-induced nausea and vomiting with acceptable side effects [53]. Aprepitant specifically exerted an anti-proliferation effect in NE-like and tNEPC tumor cells with high NK1R expression, which showed no response to enzalutamide treatment (Fig. 6A). In accordance, knockdown of NK1R demonstrated remarkable influence on tumor growth originated from these cells in vitro and in vivo, along with the reduction of the NE features expression and the signaling activity related to cell proliferation and survival (Figs. 4, 6). Chemotherapies such as taxane and platinum are efficient for patients with treatment-induced NEPC or patients with some clinical features of NEPC (NE-like prostate cancer) [54, 55], however, adverse effects like toxicity, nausea, and vomiting are serious. It was very interesting that the knockdown of NK1R greatly improved the sensitivity of 22Rv1-NE cells to docetaxel and paclitaxel therapy (Fig. S5D). Since NK1R antagonist is used to prevent chemo-induced nausea and vomiting, a regime of aprepitant combined with taxane may provide synergistic antitumor effects as well as reduced side effects. Collectively, these data suggested that antagonism of NK1R alone or in combination with other NEPC therapeutic options would be feasible and effective in tNEPC treatment.

## Materials and methods

### Cell culture and reagents

Human PCa cell line DU145, PC-3, 22Rv1, and LNCaP were purchased from American Type Culture Collection (ATCC, Manassas, VA, USA) and cultured in RPMI 1640 medium supplemented with 10% fetal bovine serum (Thermo Fisher, Waltham, USA) and 1% penicillin/streptomycin solution (Sangon, Shanghai, China) in a humidified incubator at 37 °C with 5% CO_2_. Enzalutamide (ENZ), Dihydrotestosterone (DHT), GF109203X and MLN8237 were from Selleck (Houston, TX, USA). Aprepitant was from MedChemExpress (NJ, USA). All the drugs were dissolved in dimethyl sulfoxide (DMSO) at the concentration of 100 mM, aliquoted, and stored at −20 °C. Human hemokinin-1 (hHK-1) peptide (TGKASQFFGLM-NH2) were synthesized by Fmoc solid-phase synthesis system as described before [56]. ESI-TOF mass spectrometry was performed to confirm the molecular weight of the peptide. The purity of the peptide was quantified to be >95% using reversed-phase HPLC by a C18 column as the solid phase and an H_2_O: acetonitrile gradient as the liquid phase.

### Prostate cancer tissue microarray

Tissue microarray was purchased from Superchip (HProA150PG02, Shanghai, China), containing 52 pairs of prostate cancer and adjacent tissues from 90 patients and three normal prostate tissues. The tissue microarray contained 16 paired Gleason 6 tissues, 34 paired Gleason 7 tissues, and 2 paired Gleason 8 tissues. Other information about the prostate cancer specimens was shown in Table S1.

### Generation of neuroendocrine prostate cancer cell lines

To develop prostate adenocarcinoma cell-derived NEPC cells, 22Rv1 and LNCaP cells were incubated in 1640 medium with 10% certified charcoal-stripped fetal bovine serum (CSF) (Biolind, Israel) and exposed to gradually increasing concentration of ENZ for at least 6 months passage. The established cell lines were maintained in a culture medium with 30 μM ENZ.

### Bioinformatics analysis

GSE66187, GSE3325, and GSE33316 were downloaded from the Gene Expression Omnibus (GEO) database. GSE66187 contained 20 prostate adenocarcinoma patient-derived xenografts (AR-positive/CHGA-negative) and four NEPC patient-derived xenografts (AR-negative/CHGA-positive). GSE33316 consisted of five sham-castrated and five castrated LuCap35 PDX xenografts. And GSE3325 was formed with six benign prostate tissues, seven clinically localized primary prostate cancer tissues, and six metastatic prostate cancer tissues. The dataset of Beltran et al. (2016) was obtained from the cBioPortal (http://www.cbioportal.org/). The cohort of Beltran (2016) included 34 CRPC-Adeno and 14 CRPC-derived NEPC samples. GSE16560, GSE70769, Firehose Legacy, and MSKCC prostate cancer data were used to validate the relationship between NK1R expression and patients’ survival. Data for GSE16560 and GSE70769 were obtained from the GEO database. Data on Firehose Legacy and MSKCC was obtained from the cBioPortal. A total of 491 samples (281 from GSE16560, 40 from GSE70769, 60 from Firehose Legacy, and 110 from MSKCC) was used in prostate cancer prognosis evaluation.

### Cell lines transfection

NK1R knockdown was performed as previously described [23]. The lentivirus particles containing shNK1R were obtained from OBIO Technology (Shanghai, China). The shRNA sequences were cloned into lentiviral vector pLKD-CMV-EGFP-Puro respectively (shctrl: 5´-TTCTCCGAACGTGTCACGT-3´; shNK1R 1#: 5´-GCAACCAGCCTG GCAAATT-3´; shNK1R2#: 5´-GCCTGTTCTACTGCAAGTT-3´). Cells were transfected with the lentivirus particles according to the manufacturer’s instructions followed by puromycin selection (5 μg/ml, Solarbio, Beijing, China). For NK1R overexpression, pLenti-EGFP-Puro-CMV-NK1R plasmid (OBIO) was transfected into cells with GC Liposomal Transfection Reagent (Genecarer, Xi’an, China) followed with puromycin selection.

A dual sgRNA CRISPR/Cas9 system was used to delete the ARE1 sequence in the enhancer region of the NK1R gene. Briefly, sgRNA sequences (sgRNA-F: 5’-GTACGA ATAGCCATCATATCCTGG -3’; sgRNA-R: 5’-GTCCTAAGAGCATTACACCTG AGG-3’) were cloned into the vector of Lenti-CRISPR-dual gRNA. The cells were transfected with Lenti-CRISPR-dual gRNA-ARE1 or vector control plasmid with GC Liposomal Transfection Reagent for 48 h, then harvested for the analysis of ARE1 deletion efficiency using Takara ExTaq PCR kit (Dalian, China) with the forward primer 5’-AGCGGTTTCCCAGTAGAGTC-3’ and the reverse primer 5’-AAGGGTTCAGCATGTTCTGC-3’.

### Chromatin immunoprecipitation (CHIP)

Cells were cross-linked with 1% Formaldehyde Solution (Sigma-Aldrich) for 15 min at 37 °C, quenched by 125 mM Glycine (Sangon Biotech, Shanghai, China) for 5 min at room temperature, and sonicated to shear DNA. CHIP assay was performed with CHIP Assay Kit (Beyotime) according to the manufacturer’s protocol. Chromatin fragments were precipitated with anti-AR (#51533, CST, 1:100 dilution) or a control non-immune IgG (#7074, CST, 1:100 dilution). The precipitate was eluted and analyzed by QPCR using TB Green Premix Ex TaqTM kit (Taka ra) and the Applied Biosystems QuantStudio instrument (Thermo Fisher). The primers of CHIP-QPCR were listed in Table S2.

### Real-time quantitative PCR (QPCR)

Total RNA was extracted using RNAiso Plus (Takara). About 2 μg of purified RNA was reversely transcribed into cDNA by the PrimeScriptTM RT master mix reagents (Takara). Real-time quantitative PCR was performed with TB Green Premix Ex TaqTM kit (Takara) in Applied Biosystems QuantStudio instrument (Thermo Fisher). The real-time quantitative PCR reaction was conducted with the condition: 30 s at 95 °C, 40 cycles of 5 s at 95 °C, followed by 20 s at 60 °C. All the primers was synthesized by Sangon (Shanghai, China) and listed in Table S2. The relative expression of genes was normalized by β-actin. The data were shown as –ΔΔCT.

### Co-immunoprecipitation

Co-immunoprecipitation assay was performed to evaluate the protein-protein interaction between Aurora A and N-Myc/PKCɑ. The total protein of 1 × 10^6^ cells was extracted in RIPA lysis buffer with ‘phenylmethylsulfonyl fluoride (PMSF) and 1X Phosphatase inhibitor cocktail. About 100 μg of the lysates was immunoprecipitated at 4 °C overnight with anti-N-MYC (#51705, CST), anti-Aurora A (#14475, CST), anti-PKCɑ (GTX130453, Gene Tex), or anti-IgG (#7074, CST) diluted at 1:100. The precipitate was incubated with 40 μl protein A + G agarose beads (Beyotime) at 4 °C for 3 h, then washed with cold PBS three times. The precipitates was immunoblotted with the corresponding primary antibodies and visualized in Chemiluminescence Imaging System.

### Western blot

Cells were plated and incubated with drugs as indicated. Total protein was extracted with RIPA lysis buffer supplemented with 1 mM PMSF (Sangon) and 1X phosphatase inhibitor cocktail. Total protein concentration was measured by BCA kit (Thermo Scientific) and separated by SDS-PAGE, then transferred to PVDF membranes (Millipore). The PVDF membranes were blocked with 6% skim milk (Sangon) with 0.05% Tween-20 (Sangon) at room temperature for 2 h, then incubated with the indicated primary antibodies (1:1000) overnight at 4 °C. After incubation with HRP-labeled secondary antibody (1:2000) at room temperature for 2 h, the enhanced chemiluminescence kit (NCM, Suzhou, China) was used to visualize the bands in Chemiluminescence imaging system (Cytiva, Japan). The band intensity was analyzed with Image J and normalized by GAPDH. The primary antibodies, anti-NK1R (#183713) was from Abcam; anti-AR (#51533), anti-N-MYC (#51705), anti-Aurora A (#14475), anti-pAurora A (#3079), and anti-PSA (#5365) were from Cell Signaling Technology (CST); anti-chromogranin A (CgA) (#D162602), and anti-Enolase 2 (ENO2, #161056) were from Sangon Biotech (Shanghai, China); anti-Ki67 (#51533 s) was from Sigma; anti-pPKCα (28926-1-AP) and anti-GAPDH (10494-1-AP) was from Protein Tech Group; anti-PKCα (GTX130453) was from GeneTex. The secondary antibody rabbit anti-IgG (A0208) were from Beyotime. The detailed information on antibodies and reagents was listed in Table S3.

### Cell proliferation assay

Cell proliferation was measured with a CellTiter-Glo kit according to the manufacturer’s manual (Promega, USA). About 1 × 10^3^ cells were plated in a 96-well plate and incubated with 1 μM hHK-1 for the indicated time. To measure the drug effect on cell survival, 3 × 10^3^ cells were plated in a 96-well plate and treated with the indicated drugs for 72 h. The luminescence was measured by Flex Station III microplate reader (Molecular Devices, Sunnyvale, Silicon Valley, USA). For colony formation assay, 800 cells were seeded into a six-well plate and incubated with hHK-1 in RPMI 1640 medium. The cells were replaced with fresh medium every 3 days for 2 weeks, then were fixed in 90% ethanol and stained with 0.1% crystal violet. The colonies >1 mm were counted by Image J.

### Transwell assay

About 5 × 10^4^ cells suspended in 100 μl RPMI 1640 medium were plated into the upper chamber of transwell pre-plated with Matri-Gel (Corning), then inserted in a 24-well culture plate supplemented 600 μl RPMI 1640 medium with an indicated drug. After 24 h, the invasion of cells were fixed in 90% ethanol for 30 min and stained with 0.1% crystal violet for 20 min at room temperature. Non-infiltrated cells were removed by cotton swabs. After drying, the cells were randomly photographed in three different areas and quantified using an image processing system microscope (Nikon, Japan).

### Flow cytometry

About 3 × 10^6^ cells were harvested to detect NK1R expression on the cell surface. The cells were incubated with anti-NK1R (Abcam, 1:50) for 30 min at room temperature, washed with cold PBS, and then incubated with Coralite594-conjugated goat anti-rabbit IgG (H + L) antibody (ProteinTech, 1:50) for 30 min at room temperature. Cells were washed with cold PBS three times before evaluation with BD LSRFortessa Flow Cytometer (BD Biosciences, Franklin Lake, NJ, USA) and analyzed by FlowJo software.

For cell cycle analysis, 1 × 10^6^ cells were synchronized by a double thymidine block. Cells were treated with hHK-1 or aprepitant/GF109203X/MLN8237 as indicated for 48 h, then were harvested, washed with PBS three times, and fixed with 75% ethanol at 4 °C overnight. Fixed cells were stained with 50 μg /ml PI solution containing 100 μg/ml RNAase and 0.2% triton X-100 for 30 min at 4 °C in the dark. The labeled cells were detected using BD LSRFortessa Flow Cytometer (BD Biosciences). Data were analyzed with Modfit LT 3.0 software.

### Immunohistochemistry (IHC)

IHC assay was performed to examine protein expression in human prostate cancer tissue microarray and tumor tissue section from xenografts of prostate cancer models. The sections were dewaxed with xylene and hydrated by a standard xylene-ethanol procedure, followed by antigen retrieval in 10 mM sodium citrate buffer (Solarbio). Endogenous peroxidase activity was blocked with 3% H_2_O_2_ for 10 min at room temperature. The sections were blocked with normal goat serum to block non-specific sites for 18 min at room temperature. Subsequently, the sections were incubated with the indicated primary antibodies at 4 °C overnight. HRP-conjugated secondary antibody was incubated with the sections at room temperature for 30 min. DAB (Service, Wuhan, China) was applied to visualize the protein expression followed by counterstaining nuclei with hematoxylin. For histomorphometric analysis, tissue sections were stained with hematoxylin and eosin. The sections were captured by a NIS-element imaging system (Nikon, Japan).

### Mouse tumor xenograft models

All animal experiment procedures were approved by the Ethics Committee of Animal Experimentation and conducted according to the guidelines of the Center of Medical Experiment at Lanzhou University. Male athymic nude BALB/C mice aged at 4–6 weeks were purchased from GemPharmtechnology (Jiangsu, China), and maintained in animal house with the standard specific pathogen-free (SPF) condition. The feeding environment temperature of mice is 23–25 °C, and mice can freely ingest food and water. About 2 × 10^6^ cells of each cell line were suspended in a 100 μl mixture of Matrigel matrix and PBS (1:2) and injected into the right flank of nude mice. Tumor volume was measured every 2 days after the injection. The tumor size was calculated according to the following formula: tumor volume = 1/2 × length (mm) × width (mm)^2^. At the indicated time point, the mice were euthanized and the tumors isolated. Tumor weight was recorded. Tumor tissue was fixed in 4% formalin and embedded in paraffin for tissue sections or retained at −80 °C.

### Statistical methods

Data from at least three independent experiments were shown as the mean ± standard deviation (SD) in GraphPad Prism 7.00. One-way ANOVA with Tukey’s test and two-tailed Student’s *t*-test was used to calculate the statistical significance between different groups. Correlation analysis was performed to evaluate the correlation between NK1R expression level and the Gleason grade of prostate cancer. Log-rank test was used in Kaplan–Meier analysis. The difference is statistically significant as follows: P* < 0.05, P** < 0.01, P*** < 0.001.

## Supplementary information

aj-checklist

Supplementary Figure 1

Supplementary Figure 2

Supplementary Figure 3

Supplementary Figure 4-1

Supplementary Figure 4-2

Supplementary Figure 5

Supplementary Figure Legends and tables

Full and uncropped western blots

## Data Availability

The accession number for all raw sequencing data are available in GEO under accession numbers: GSE16560, GSE70769, GSE33316, GSE66187, and GSE3325. The dataset of Beltran et al. (2016), Firehose Legacy, and MSKCC prostate cancer data were obtained from the cBioPortal (http://www.cbioportal.org/). Most important materials and products are listed in supplementary Tables S1–S3.
