# Supplementary figures and images for: Neurokinin-1 receptor drives PKCɑ-AURKA/N-Myc signaling to facilitate the neuroendocrine progression of prostate cancer
Source: Cell Death Dis. 2023 Jun 29;14(6):384. doi: 10.1038/s41419-023-05894-x (PMC10310825; doi:10.1038/s41419-023-05894-x)

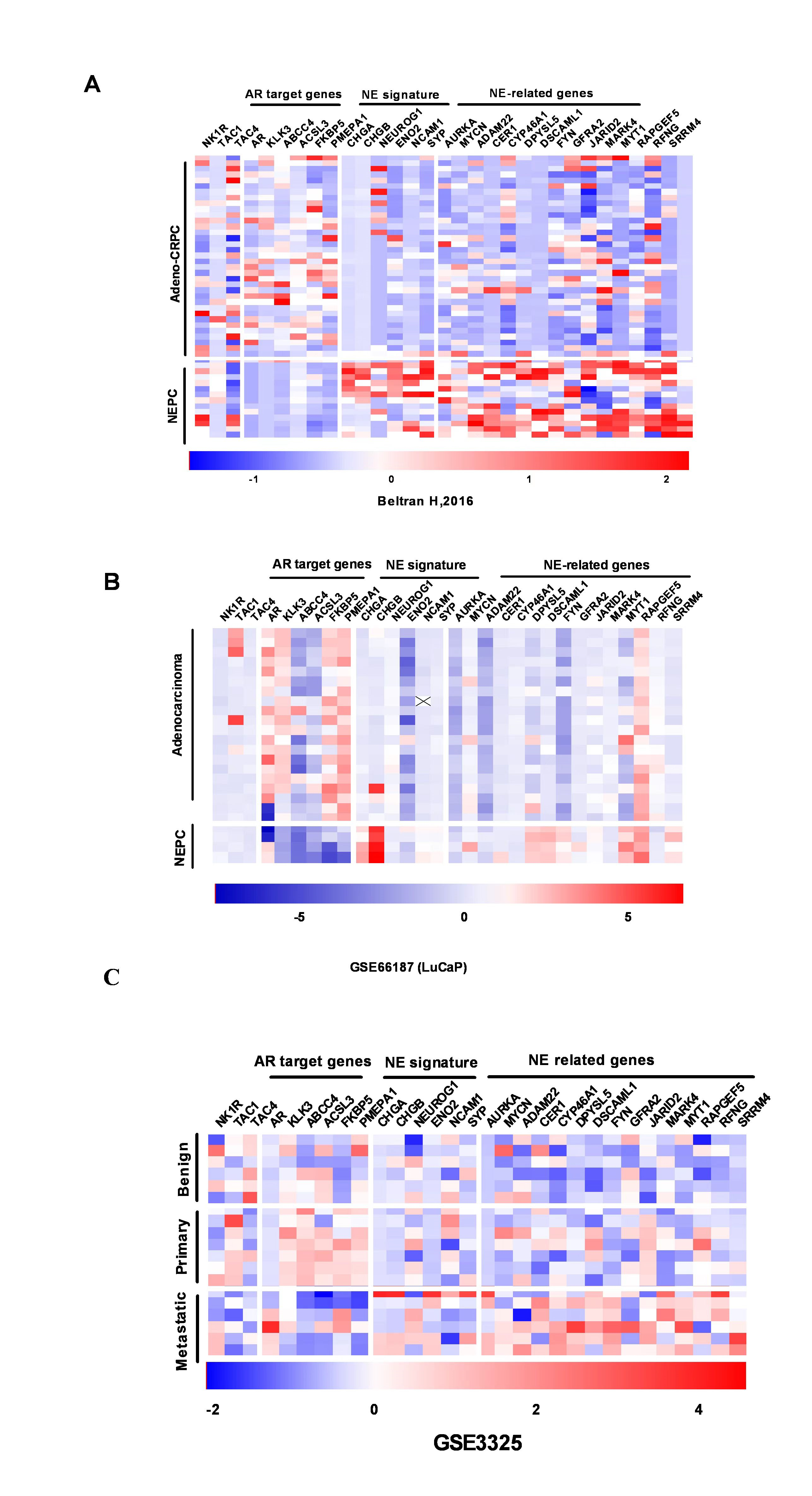

Supplement: Supplementary file 2 — Supplementary Figure 1 [file 41419_2023_5894_MOESM2_ESM.png]

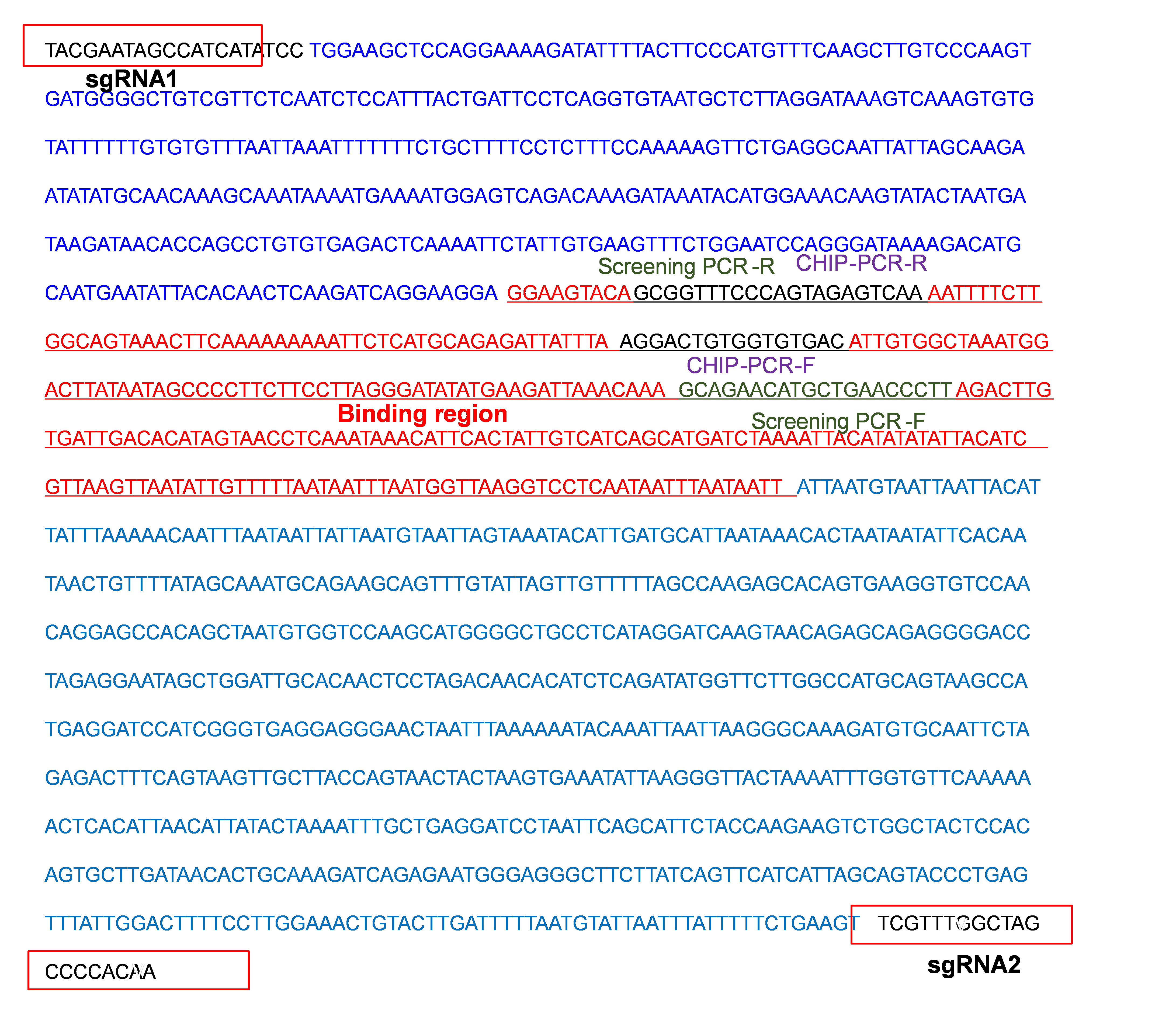

Supplement: Supplementary file 3 — Supplementary Figure 2 [file 41419_2023_5894_MOESM3_ESM.png]

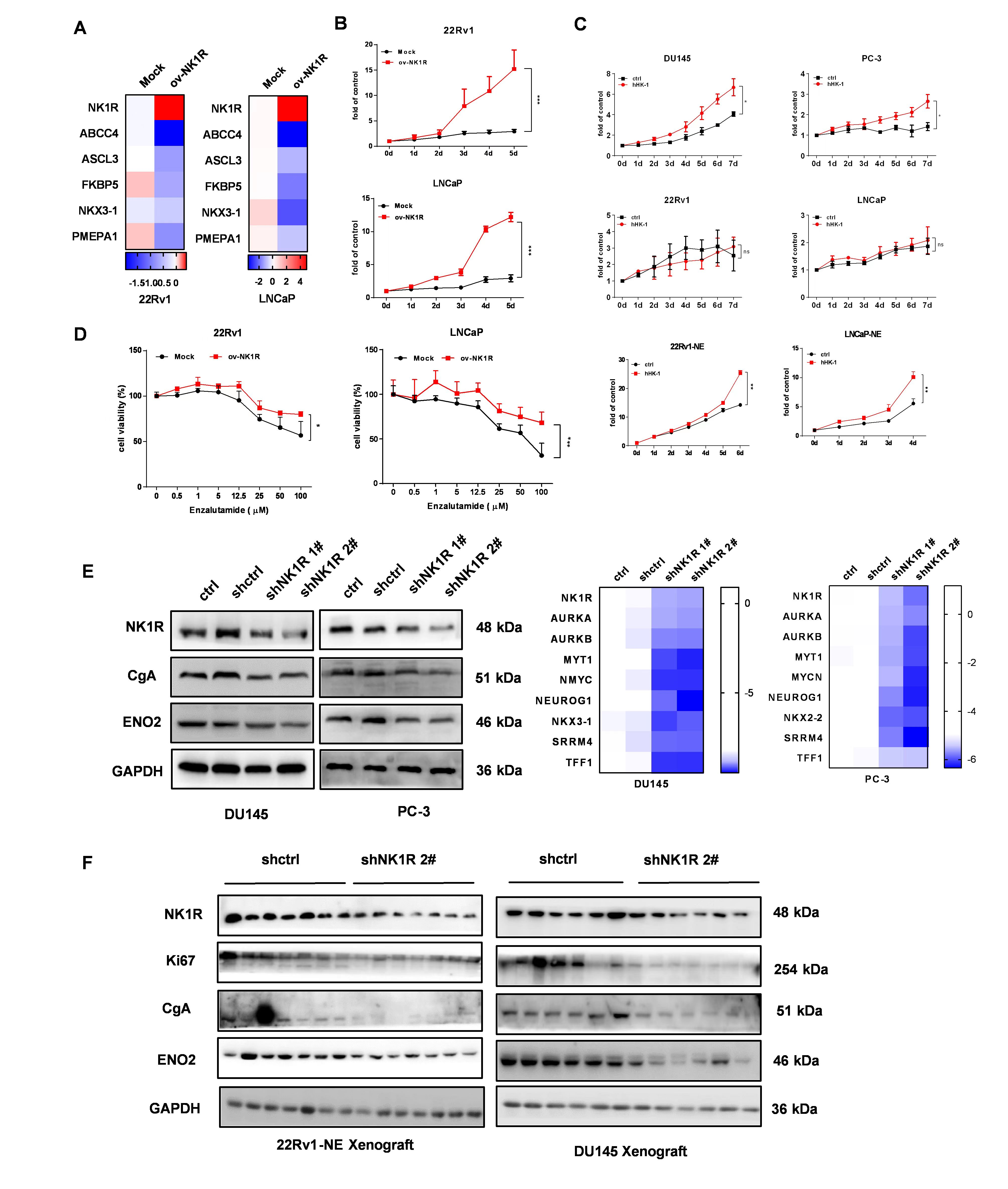

Supplement: Supplementary file 4 — Supplementary Figure 3 [file 41419_2023_5894_MOESM4_ESM.png]

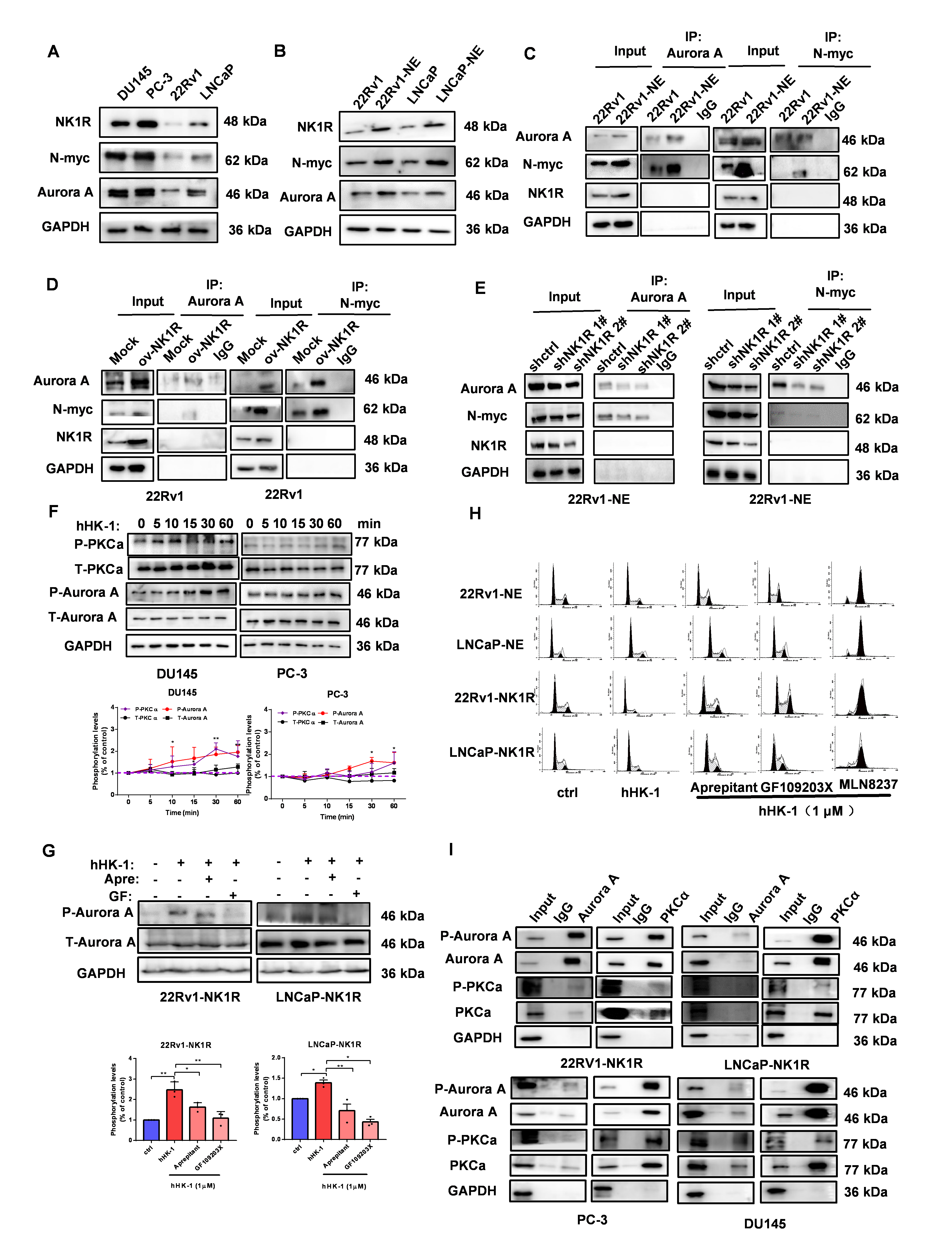

Supplement: Supplementary file 5 — Supplementary Figure 4-1 [file 41419_2023_5894_MOESM5_ESM.png]

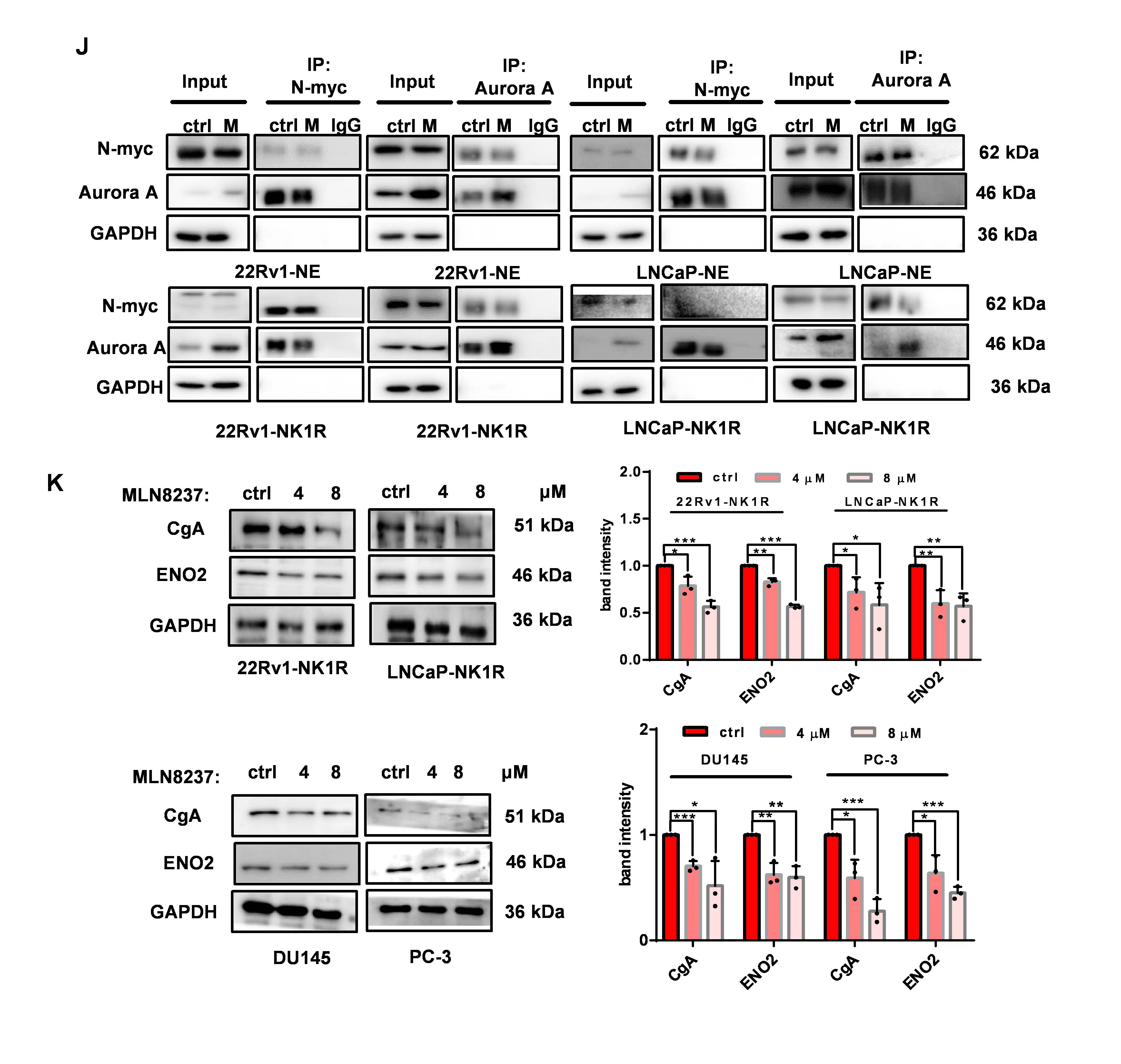

Supplement: Supplementary file 6 — Supplementary Figure 4-2 [file 41419_2023_5894_MOESM6_ESM.png]

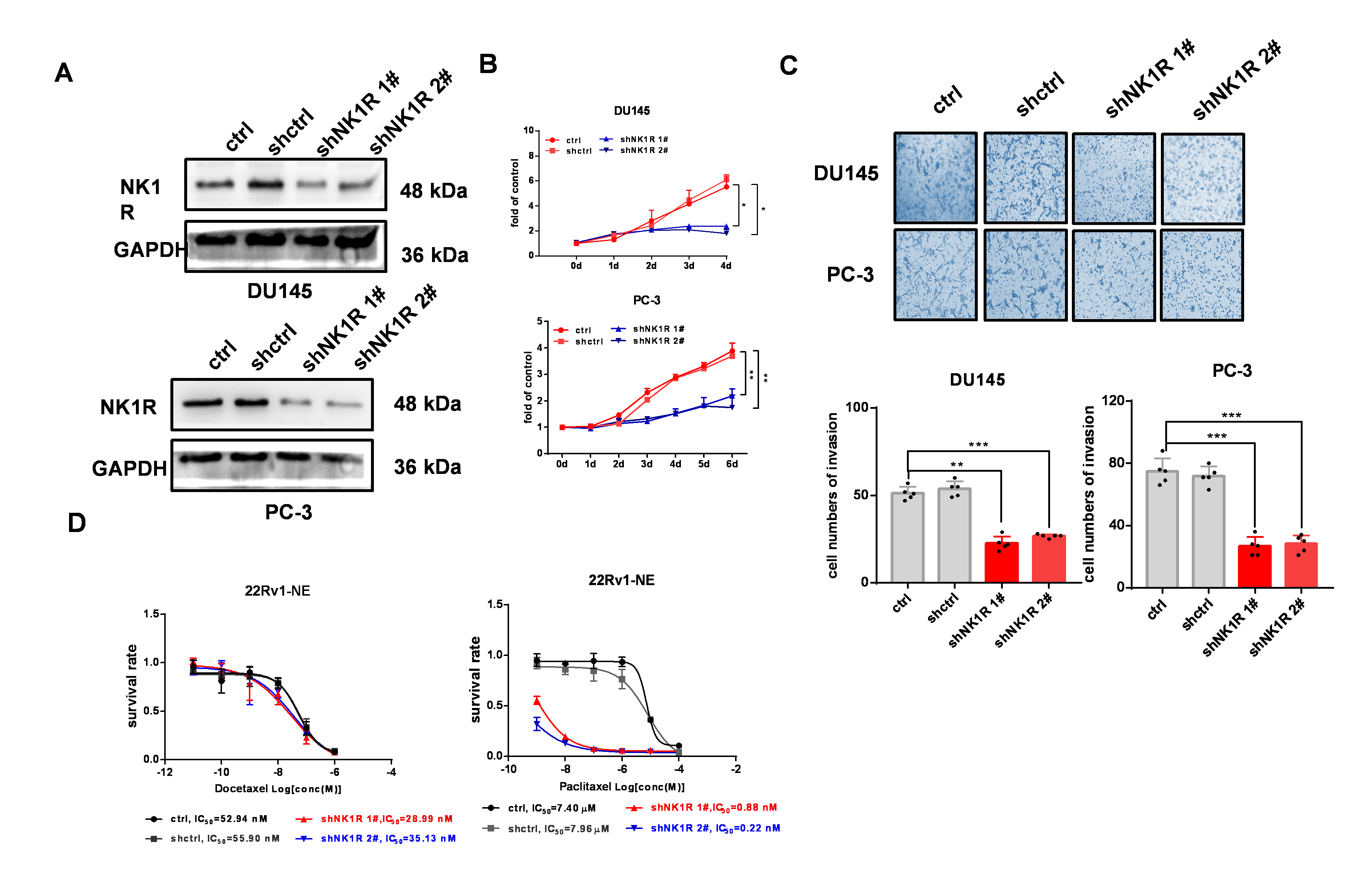

Supplement: Supplementary file 7 — Supplementary Figure 5 [file 41419_2023_5894_MOESM7_ESM.png]
