# Supplementary material for: Neurokinin-1 receptor drives PKCɑ-AURKA/N-Myc signaling to facilitate the neuroendocrine progression of prostate cancer
Source: Cell Death Dis. 2023 Jun 29;14(6):384. doi: 10.1038/s41419-023-05894-x (PMC10310825; doi:10.1038/s41419-023-05894-x)
Supplement: Supplementary file 8 — Supplementary Figure Legends and tables [file 41419_2023_5894_MOESM8_ESM.docx]

**Supplementary information for**

**Neurokinin-1 receptor drives PKCɑ-AURKA/N-Myc signaling to facilitate the neuroendocrine progression of prostate cancer**

**Authors**

Xiao-Wei Zhang, Jing-Yi Li, Lin Li, Wen-Qian Hu, Yan Tao, Wen-Yan Gao, Zi-Nuo Ye, Hao-Yuan Jia, Jia-Nan Wang, Xiao-Kang Miao, Wen-Le Yang Rui Wang^*^, Ling-Yun Mou^*^

**This PDF file includes:**

**1. Supplementary figure legends**

**2. Table 1-3**

**1. Supplementary figure legends**

**FigS.1** Heatmaps of AR target genes (left), NE signature (middle), NE-related genes (right), and NK1R in human prostate cancer samples in (**A**) Beltran H et al. 2016, (**B**) GSE66187 and (**C**) GSE3325.

**FigS.2** The sequence of AR response element (ARE1) and dual sgRNA targeting sites on NK1R gene ending site.

**FigS.3 A** mRNA level of AR target genes in 22Rv1/LNCaP cells transfected with ov-NK1R plasmids. **B** Comparison of the effect of NK1R overexpression on growth curve in 22Rv1 and LNCaP cells. **C** The effect of NK1R activation by 1 μM hHK-1 on growth curve in DU145/PC-3, 22Rv1/LNCaP and 22Rv1-NE /LNCaP-NE cells. **D** NK1R overexpression increased drug resistance to ENZ treatment in 22Rv1 and LNCaP cells. The cells were treated with ENZ at indicated concentrations for 72 h and measured with CellTiter-Glo assay. **E** The effect of shRNA-mediated NK1R knockdown on CgA, ENO2 (left) and NE signature genes expression (right) in DU145 and PC-3. **F** Expression of CgA, ENO2 and proliferative marker Ki67 in tumor xenografts using western blot.

**FigS.4** The Expression of AURKA and N-myc in (**A**) various prostate cancer cell lines and (**B**) 22Rv1-NE/LNCaP-NE cells. Co-IP assay by the antibodies of N-Myc or AURKA to detect AURKA/N-Myc interaction in (**C**) 22Rv1-NE and (**D**) 22Rv1-NK1R cells. **E** The effect of NK1R knockdown on AURKA/N-Myc interaction in 22Rv1-NE cells detected by co-immunoprecipitation assay. IgG was used as negative control antibody. **F** hHK-1 induced AURKA phosphorylation in DU145/PC-3 cells in a time-dependent way. Band intensity was analyzed by image J and normalized by GAPDH (down). **G** AURKA phosphorylation level was inhibited by Aprepitant and GF109203X treatment in 22Rv1-NK1R/LNCaP-NK1R cells. The cells were pre-treated with aprepitant (2 μM) or GF109203X (1 μM) for 30 min and stimulated by 1μM hHK-1 for 30 min before subjecting to Western blot analysis. Band intensity was analyzed by image J and normalized by GAPDH (down). **H** FACS analysis of cell cycle in 22Rv1-NE/NK1R and LNCaP-NE/NK1R cells. The cells were treated with hHK-1in presence or absence of aprepitant (2 μM), GF109203X (1 μM) or MLN8237 (4 μM) for 48 h. Untreated cells were used as control. **I** Co-IP assay by the antibodies of PKCɑ or AURKA to detect PKCɑ/ AURKA interaction in DU145/PC-3 and 22Rv1-NK1R/LNCaP-NK1R cells. IgG was used as negative control antibody. **J** Co-IP assay by the antibodies of N-Myc or AURKA to detect AURKA/N-Myc interaction in cells treated by MLN8237 for 24 h. Whole cell lysate protein was used in the input group; IgG antibody was used as the negative control. **K** MLN8237 reduced the protein level of CgA and ENO2 in DU145/PC-3 and 22Rv1-NK1R/LNCaP-NK1R cells. Cells were treated with MLN8237 for 24 h and the whole cell lysates were analyzed by Western blot. Band intensity was calculated by Image J and normalized by GAPDH (right). Data was shown as mean ± SD, *P < 0.05, **P < 0.01, ***P < 0.001.

**FigS.5 A** Western blot assay of shRNA-mediated NK1R knockdown in DU145 and PC-3 cells. The effect of NK1R knockdown on (**B**) cell growth curve and (**C**) transwell cell invasion in DU145 and PC-3 cells, the infiltrated cells were calculated by Image J (down). (**D**) IC_50_ value of docetaxel and paclitaxel in 22Rv1-NE cells. Cell survival in NK1R knockdown 22Rv1-NE cells treated with docetaxel or paclitaxel as indicated for 72 h using Celltiter-glo assay. Data was shown as mean ± SD, *P < 0.05, **P < 0.01, ***P < 0.001.

**2. Supplementary table**

**Table S1: Prostate cancer TMA information.**

| **Clinic pathologic** | **Number** |
| --- | --- |
| **Age >70** | 50 |
| **Age ≤70** | 40 |
| **AR positive** | 82 |
| **AR negative** | 3 |
| **PSA positive** | 82 |
| **PSA negative** | 3 |
| **Ki67 (%) >10** | 12 |
| **Ki67 (%) ≤10** | 74 |
| **P53 positive** | 14 |
| **P53 negative** | 72 |

**Table S2: Primers in this study.**

| **Name** | **Sequence** | **Application** |
| --- | --- | --- |
| NK1R-F | TGCTGCCTCAATGACAGGTGA | qRT-PCR |
| NK1R-R | CATTTAGGATGGCCGCTTGGC | qRT-PCR |
| AR-F | ATGGTGAGCAGAGTGCCCTATC | qRT-PCR |
| AR-R | ATGGTCCCTGGCAGTCTCCAAA | qRT-PCR |
| KLK3-F | CGCAAGTTCACCCTCAGAAGGT | qRT-PCR |
| KLK3-R | GACGTGATACCTTGAAGCACACC | qRT-PCR |
| CHGA-F | GGTTCTTGAGAACCAGAGCAGC | qRT-PCR |
| CHGA-R | GCTTCACCACTTTTCTCTGCCTC | qRT-PCR |
| NSE-F | CTGTATCGCCACATTGCTCAGC | qRT-PCR |
| NSE-R | AGCTTGTTGCCAGCATGAGAGC | qRT-PCR |
| AURKA-F | GCAACCAGTGTACCTCATCCTG | qRT-PCR |
| AURKA-R | AAGTCTTCCAAAGCCCACTGCC | qRT-PCR |
| AURKB-F | GGAGTGCTTTGCTATGAGCTGC | qRT-PCR |
| AURKB-R | GAGCAGTTTGGAGATGAGGTCC | qRT-PCR |
| NMYC-F | ACCACAAGGCCCTCAGTACCTC | qRT-PCR |
| NMYC-R | TGACAGCCTTGGTGTTGGAGGA | qRT-PCR |
| MYT1-F | TTCAGACCAGCGAAACCTCACC | qRT-PCR |
| MYT1-R | TGTGGCTTCGTGCTGAGGTTCT | qRT-PCR |
| NEUROG1-F | GCCTCCGAAGACTTCACCTACC | qRT-PCR |
| NEUROG1-R | GGAAAGTAACAGTGTCTACAAAGG | qRT-PCR |
| NKX2-2-F | CCTTCTACGACAGCAGCGACAA | qRT-PCR |
| NKX2-2- R | ACTTGGAGCTTGAGTCCTGAGG | qRT-PCR |
| TFF1-F | CCAGTGTGCAAATAAGGGCTGC | qRT-PCR |
| TFF1-R | AGGCAGATCCCTGCAGAAGTGT | qRT-PCR |
| SRRM4-F | TGGCAACACCTCTGATTCAGGG | qRT-PCR |
| SRRM4-R | ACTTCGGCACATTCCAGACACG | qRT-PCR |
| ABCC4-F | CTGTTGGAGGATGGTGATCTGAC | qRT-PCR |
| ABCC4-R | CTGCTAACTTCCGCATCTACTGC | qRT-PCR |
| FKBP5-F | GCGAAGGAGAAGACCACGACAT | qRT-PCR |
| FKBP5-R | TAGGCTTCCCTGCCTCTCCAAA | qRT-PCR |
| PMEPA1-F | CTGAGCCACTACAAGCTGTCTG | qRT-PCR |
| PMEPA1-R | GGATTCCGTTGCCTGACACTGT | qRT-PCR |
| NKX3-1-F | CGCAGAACGACCAGCTGAGCA | qRT-PCR |
| NKX3-1-R | CCTGAAGTGTTTTCAGAGTCCAAC | qRT-PCR |
| ACSL3-F | CTTTCTCACGGATGCCGCATTG | qRT-PCR |
| ACSL3-R | CTGCTGCCATCAGTGTTGGTTTC | qRT-PCR |
| ACTB-F | CACCATTGGCAATGAGCGGTTC | qRT-PCR |
| ACTB- R | AGGTCTTTGCGGATGTCCACGT | qRT-PCR |
| ARE1-F | GCGGTTTCCCAGTAGAGTCAA | CHIP-PCR |
| ARE1-R | ACAATGTCACACCACAGTCCT | CHIP-PCR |
| ARE2-F | CTACTTGCCGTGGGAGTCTG | CHIP-PCR |
| ARE2-R | GGACCGCATCTGTGTCAGTT | CHIP-PCR |
| ARE3-F | TGGGCCCCGAGAAACTACTA | CHIP-PCR |
| ARE3-R | GGGGCTAAGATGGAATGGGG | CHIP-PCR |
| ARE4-F | GAGGAAAGACGGCTAACGGA | CHIP-PCR |
| ARE4-R | CTCAGCACACAAAGAAAGCCAA | CHIP-PCR |
| PSA enhancer-F | GAATCGGGGATCGTACCCAC | CHIP-PCR |
| PSA enhancer-R | GCCCTGTAGCTCATGGAGAC | CHIP-PCR |
| KIAA0066-F | CTAGGAGGGTGGAGGTAGGG | CHIP-PCR |
| KIAA0066-R | GCCCCAAACAGGAGTAATGA | CHIP-PCR |
| ARE1-F | AGCGGTTTCCCAGTAGAGTC | Screening PCR |
| ARE1-R | AAGGGTTCAGCATGTTCTGC | Screening PCR |
| ACTB-F | TGGTGAGCTGCGAGAATAGC | Screening PCR |
| ACTB-R | TCCGACCAGTGTTTGCCTTT | Screening PCR |
| ARE1-1 | GTACGAATAGCCATCATATCCTGG | sgRNA |
| ARE1-2 | GTCCTAAGAGCATTACACCTGAGG | sgRNA |
| shctrl | TTCTCCGAACGTGTCACGT | shRNA |
| shNK1R 1# | GCAACCAGCCTGGCAAATT | shRNA |
| shNK1R 2# | GCCTGTTCTACTGCAAGTT | shRNA |

**Table S3: antibodies and reagents resources**

| **Name** | **Manufacturer** | **Catalog number** |
| --- | --- | --- |
| Rabbit anti-NK1R | Abcam, UK | Cat#: ab183713 |
| Rabbit anti-AR | Cell Signaling Technology, Germany | Cat#: 5153 |
| Rabbit anti-PSA | Cell Signaling Technology, Germany | Cat#: 5365 |
| Rabbit anti-N-MYC | Cell Signaling Technology, Germany | Cat#: 51705 |
| Rabbit anti-Aurora A | Cell Signaling Technology, Germany | Cat#: 14475 |
| Rabbit anti-pAurora A | Cell Signaling Technology, Germany | Cat#: 3079 |
| Rabbit anti-chromogranin A | Sangon Biotech, China | Cat#: D162602 |
| Rabbit anti-Enolase | Sangon Biotech, China | Cat#: D161056 |
| Rabbit anti-PKCα | Gene Tex, USA | Cat#:GTX130453 |
| Rabbit anti-pPKCα | ProteinTech, Wuhan, China | Cat#:28926-1-AP |
| Rabbit anti-pERK | Cell Signaling Technology, Germany | Cat#: #4370 |
| Rabbit anti-pAKT | Cell Signaling Technology, Germany | Cat#: #4060 |
| Mouse anti-Ki67 | Sigma, USA | Cat#: 51533s |
| Rabbit anti-GAPDH | ProteinTech, Wuhan, China | Cat#: 10494-1-AP |
| Rabbit anti-IgG | Cell Signaling Technology, Germany | Cat#: #7074 |
| CoraLite594 - conjugated Goat Anti-Rabbit IgG(H+L) | ProteinTech, Wuhan, China | Cat#: SA0013-4 |
| Aprepiant | MedChemExpress, USA | Cat#: HY10052 |
| MLN8237(Alisertib) | Selleck Chemicals, USA | Cat#: S1133 |
| Enzalutamide | Selleck Chemicals, USA | Cat#: S1250 |
| GF109203X | Selleck Chemicals, USA | Cat#: S7208 |
| BCA kit | Thermo Scientific, Austria | Cat#: 23227 |
| Crystal violet | Sangon, China | Cat#: A100528 |
| Polybrene | Solarbio, China | Cat#: H8761 |
| Puromycin | Solarbio, China | Cat#: P8230 |
| Lipofectamine® 2000 Reagent | Invitrogen, USA | Cat#: 2357812 |
| RNAiso Plus | Takara, Japan | Cat#: 9109 |
| PrimeScript RT master mix | Takara, Dalian, China | Cat#: RR036A |
| SYBR Green Master Mix | Yeasen Biotech, Shanghai, China | Cat#: 11202ES08 |
| RIPA | Beyotime, Shanghai, China | Cat#: P0013B |
| PMSF | Sangon, Shanghai, China | Cat#: A100754 |
| Phosphatase inhibitor cocktail | Beyotime, Shanghai, China | Cat#: P1046 |
| PVDF membrane | Merck Millipore, USA | Cat#: IPVH00010 |
| ECL kit | Yeasen Biotech, Shanghai, China | Cat#: 36222ES76 |
| A+G agarose beads | Beyotime, Shanghai, China | Cat#: P2055 |
| CHIP Assay Kit | Beyotime, Shanghai, China | Cat#: P2078 |
| DAB staining kit | Service, Wuhan, China | Cat#: G1212 |
| Celltiter-Glo Cell Viability kit | Promega, USA | Cat#: G9242 |
| GC Liposomal Transfection Reagent | Genecarer, Xi’an, China | Cat#:GC1000146 |
| ExTaq PCR kit | Takara, Dalian, China | Cat#: RR001A |
