# Supplementary material for: Neurokinin-1 receptor drives PKCɑ-AURKA/N-Myc signaling to facilitate the neuroendocrine progression of prostate cancer
Source: Cell Death Dis. 2023 Jun 29;14(6):384. doi: 10.1038/s41419-023-05894-x (PMC10310825; doi:10.1038/s41419-023-05894-x)

**Full and uncropped western blots**

**Neurokinin-1 receptor drives PKCɑ-AURKA/N-Myc signaling to facilitate the neuroendocrine progression of prostate cancer**

**Authors**

Xiao-Wei Zhang, Jing-Yi Li, Lin Li, Wen-Qian Hu, Yan Tao, Wen-Yan Gao, Zi-Nuo Ye, Hao-Yuan Jia, Jia-Nan Wang, Xiao-Kang Miao, Wen-Le Yang Rui Wang^*^, Ling-Yun Mou^*^

**
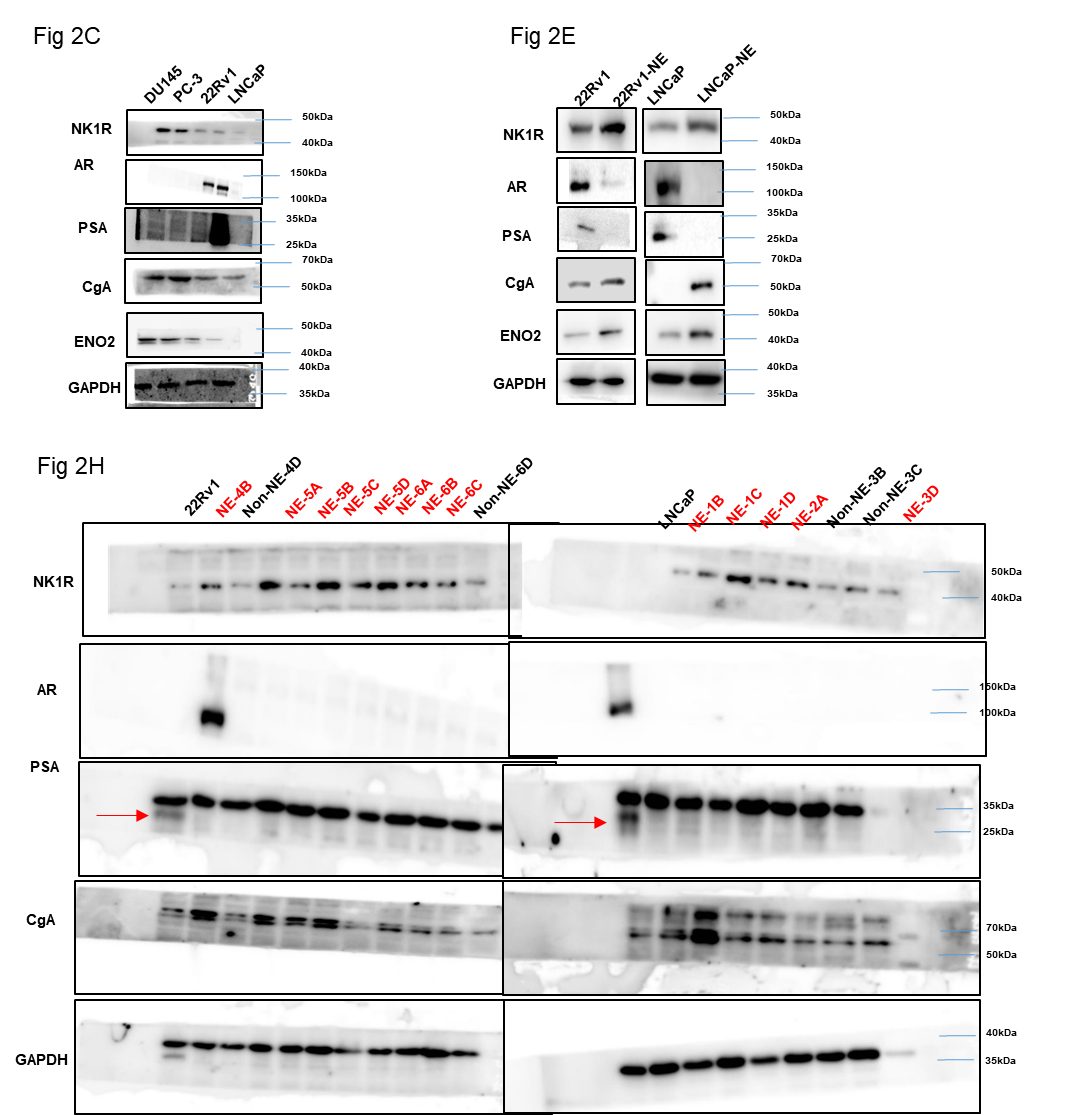
**


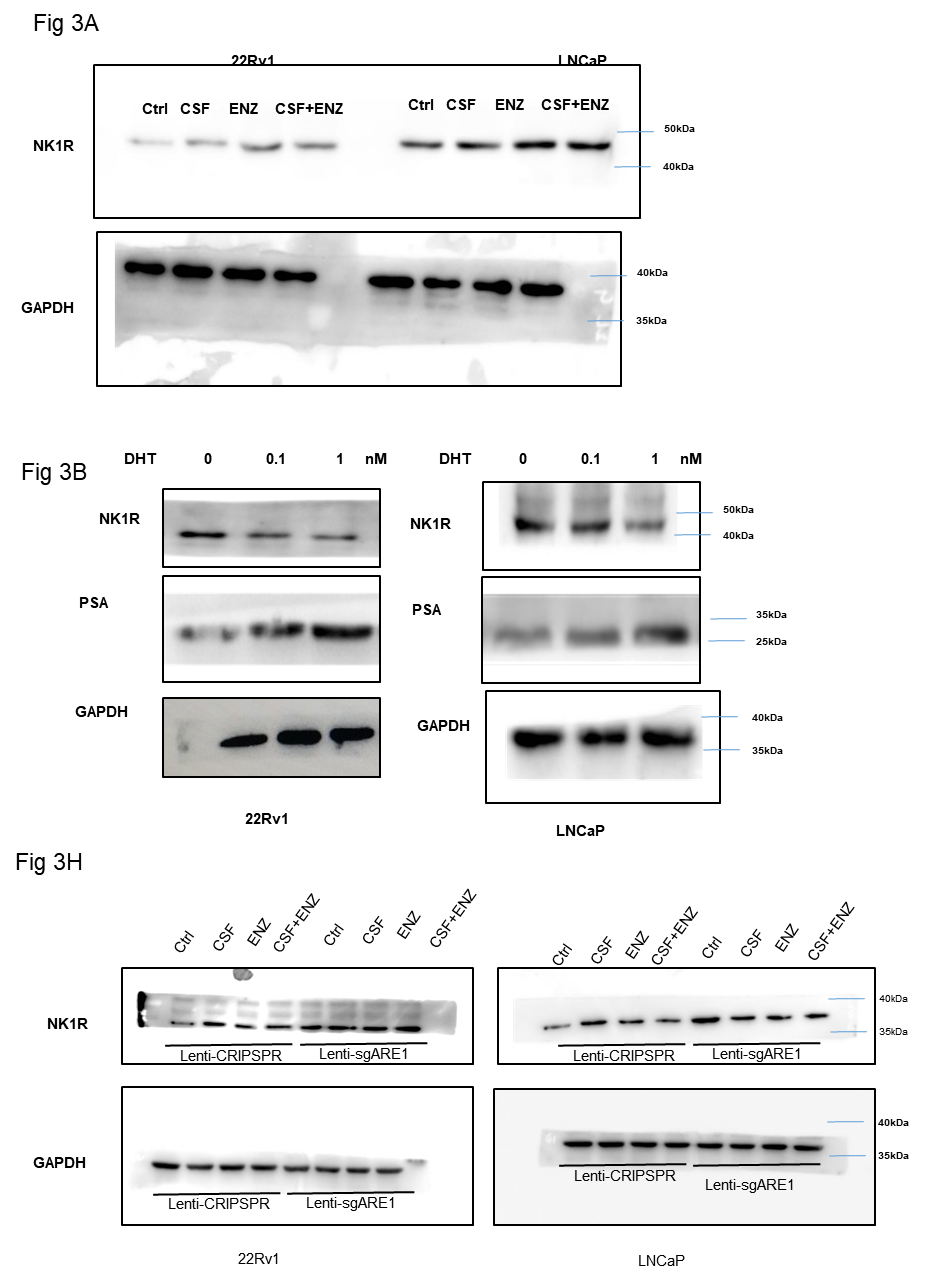


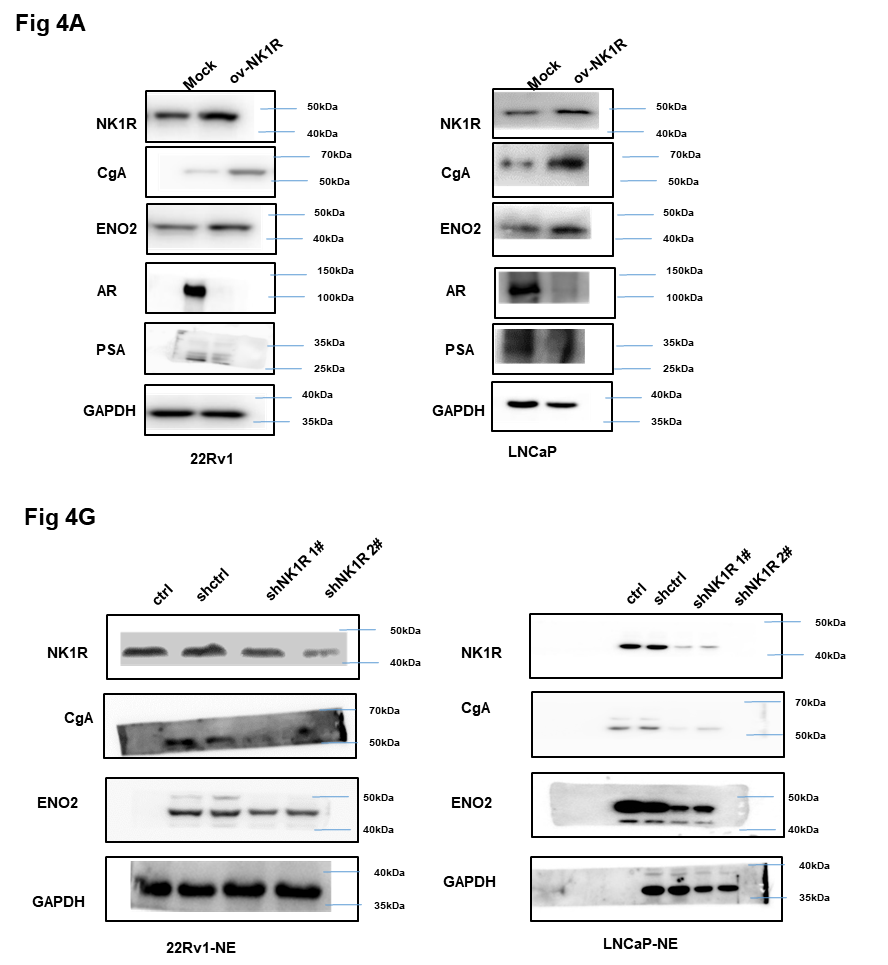


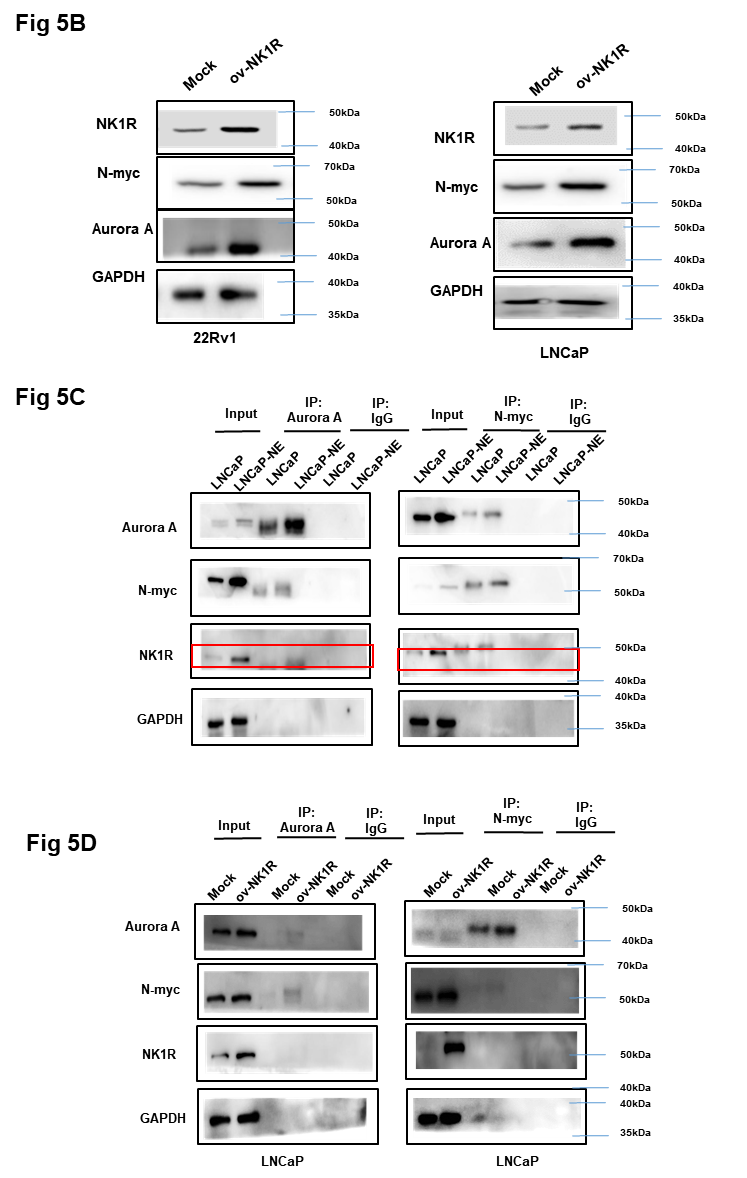


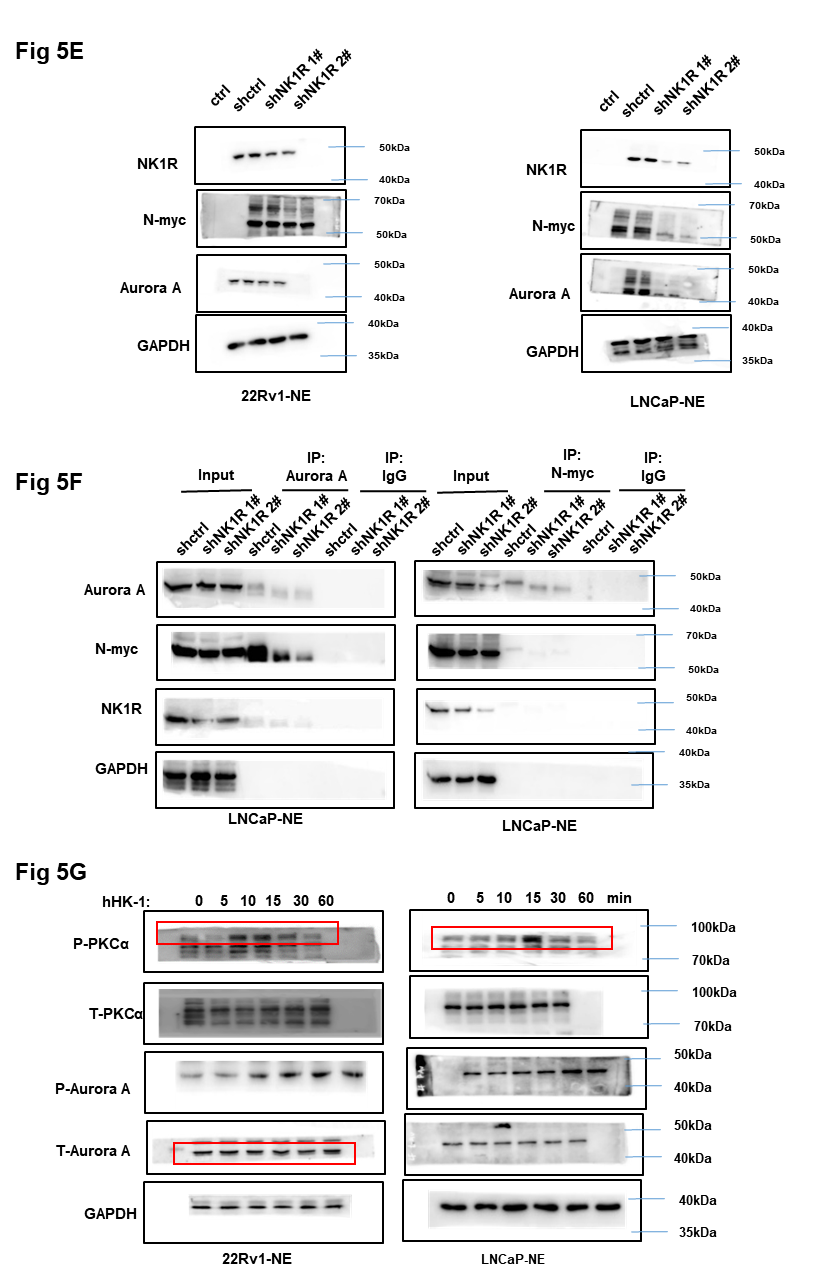


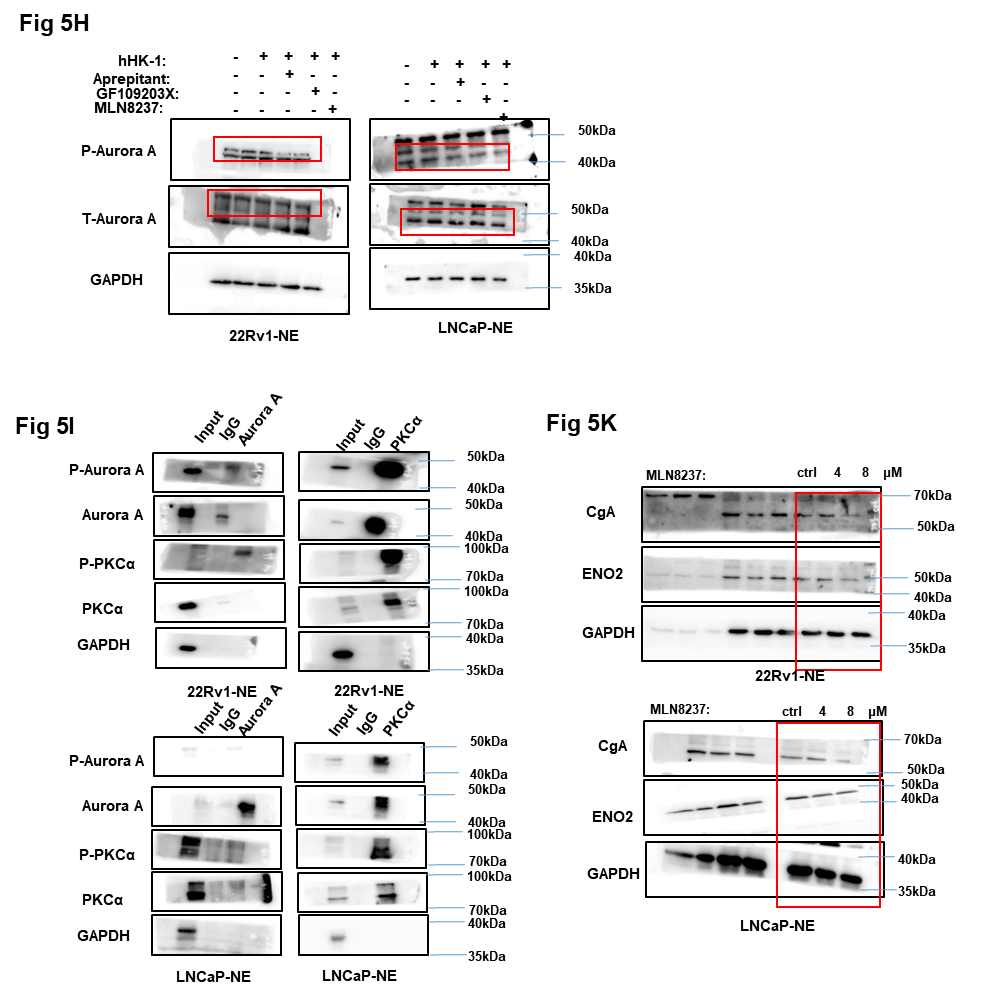


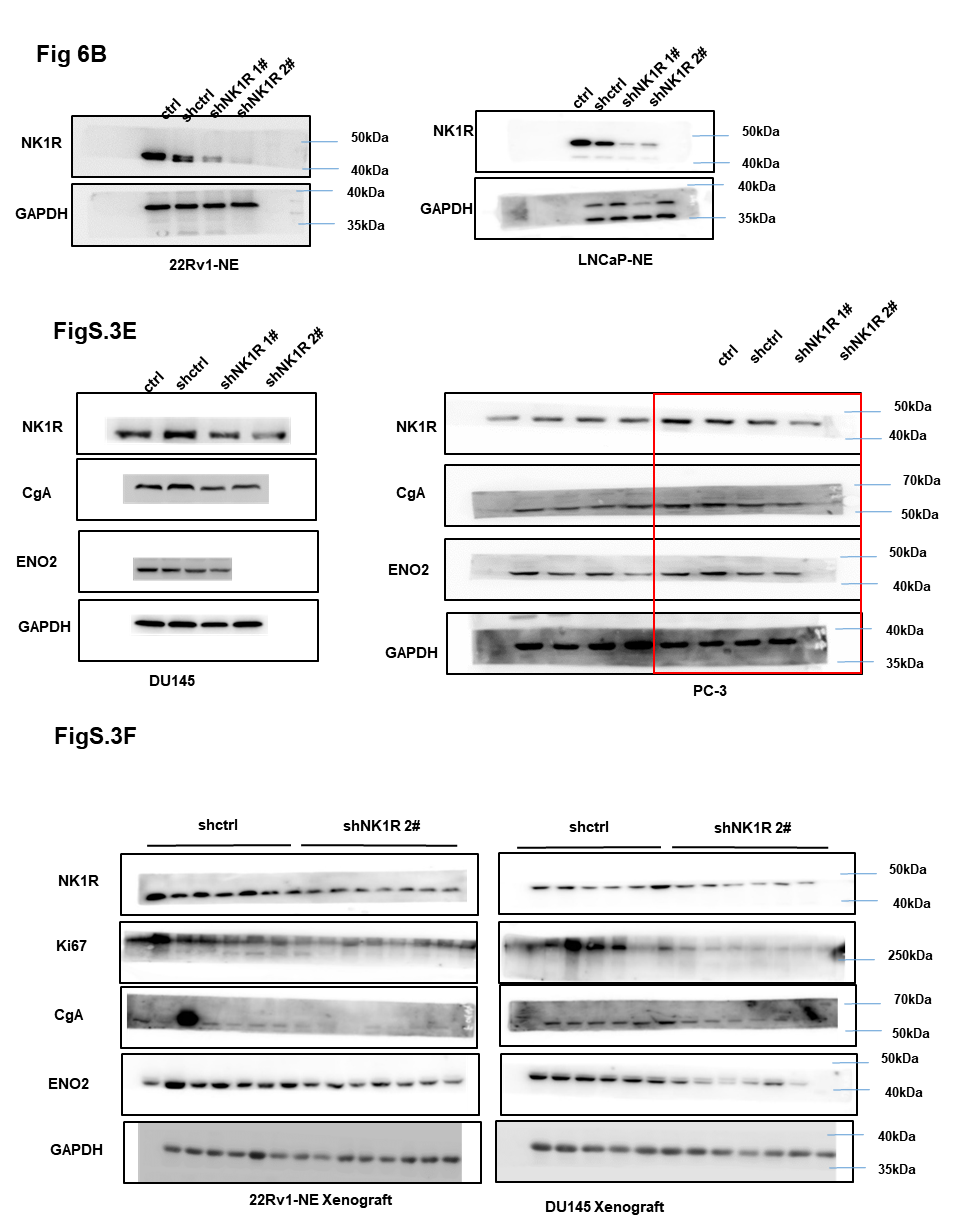


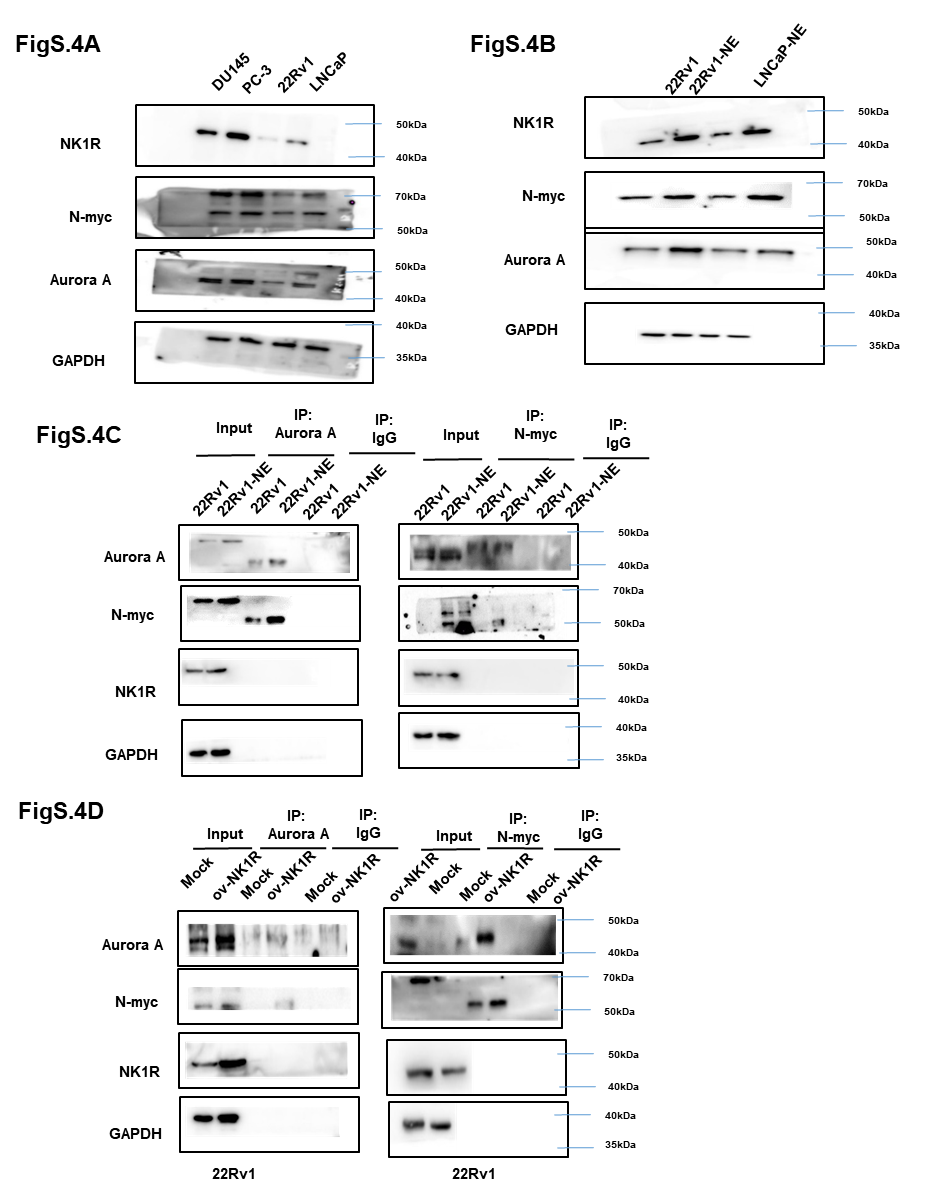


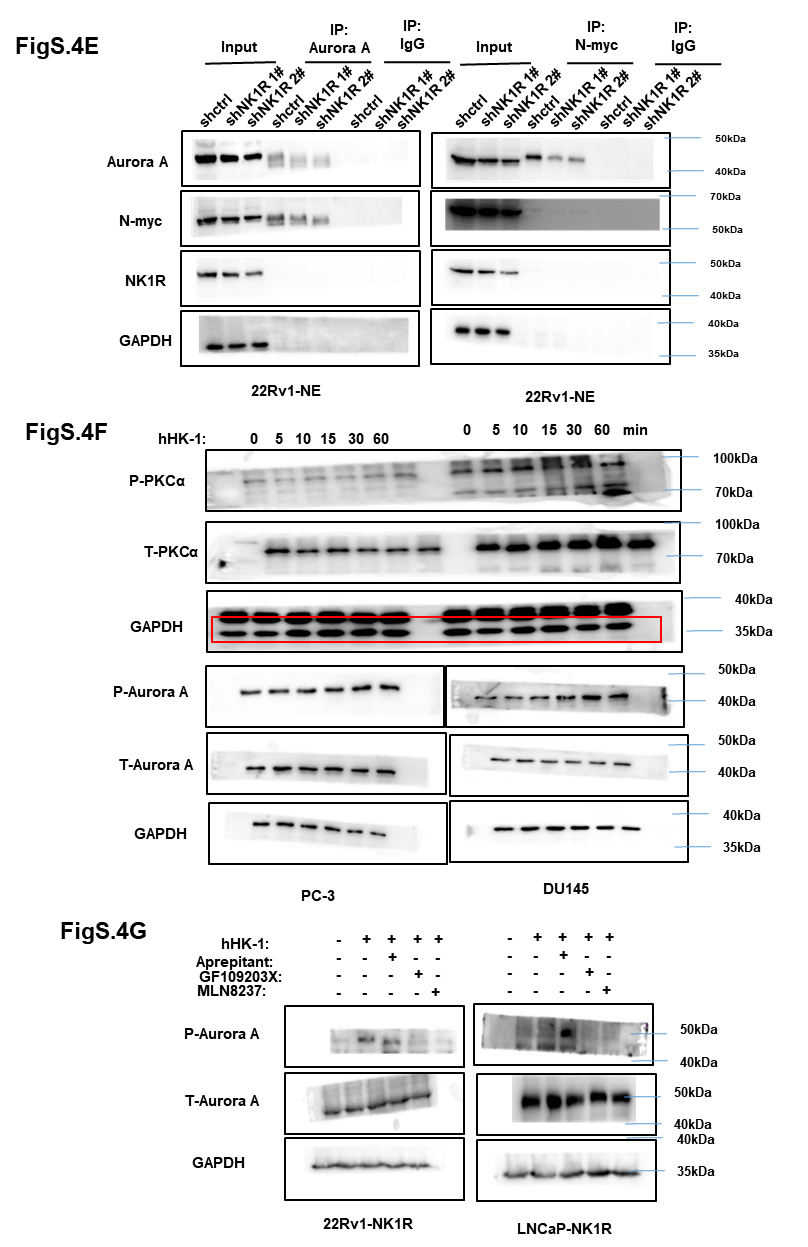


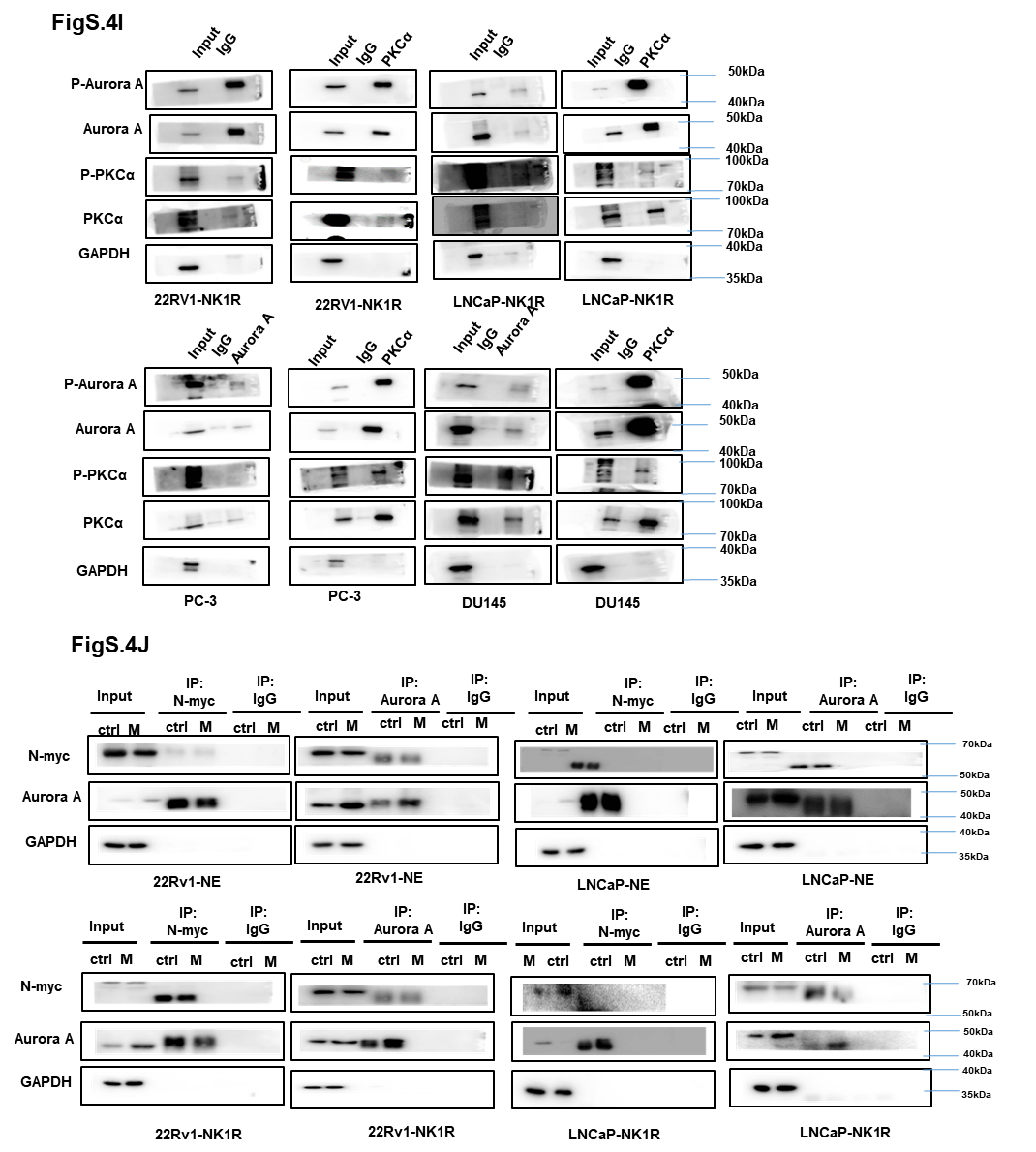


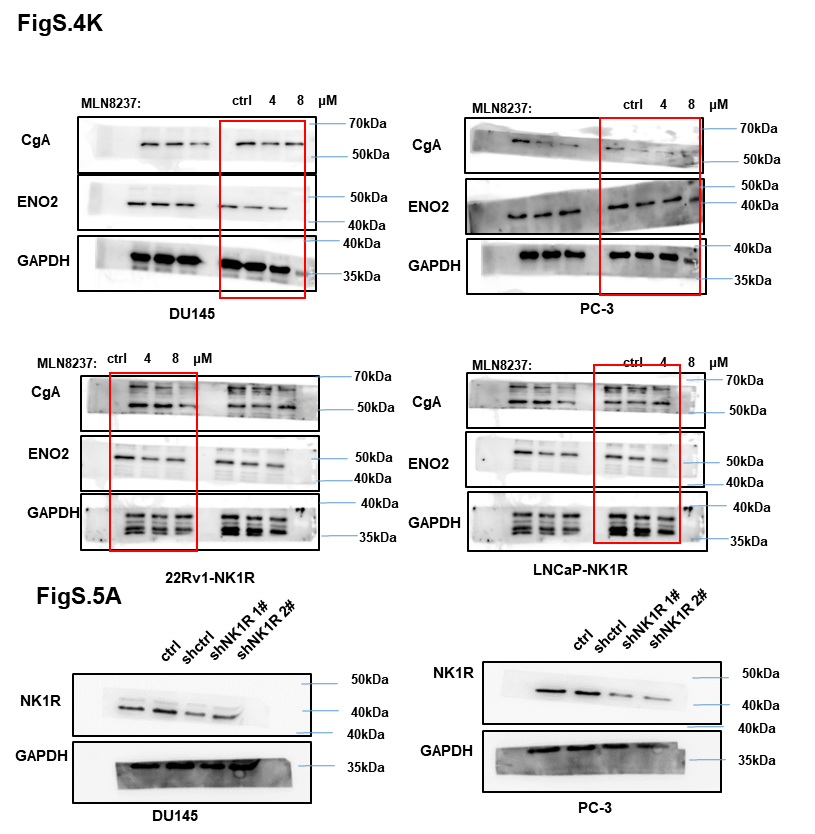

Supplement: Supplementary file 9 — Full and uncropped western blots [file 41419_2023_5894_MOESM9_ESM.docx]
